# Thermal-structural hybrid Lagrangian solver and numerical simulation-based correction of shape deformation of stainless-steel parts produced by laser powder bed fusion

**DOI:** 10.1038/s41598-023-43968-0

**Published:** 2023-10-16

**Authors:** Ilya Tsivilskiy, Igor Shishkovsky

**Affiliations:** 1 Kazan Federal University, Kazan, Russian Federation 420008; 2 Center for Materials Technologies, Skolkovo Institute of Science and Technology, Moscow, Russian Federation 121205

**Keywords:** Mechanical engineering, Computational methods, Mechanical properties

## Abstract

An efficient thermal-structural numerical solver for Additive Manufacturing has been developed based on a modified Lagrangian approach to solve the energy conservation equations in differential form. The heat transfer is modeled using the finite difference method applied to a deforming Lagrangian mesh. The structural solver has been enhanced with the proposed effective quasi-elastic differential approach for modeling the elastoplastic behavior of materials. The algorithm is relatively simple to implement yet is highly effective. The solver can predict shape deformations of metal parts printed using the laser powder bed fusion technique. The second key capability of the solver is the auto-compensation of distortions of 3D-printed parts by proposing a corrected geometry of a surface to be printed, in order to ensure minimal deviation of the actual printed part from the desired one, even under non-optimal operating conditions or for complex shapes. All the simulation results have been verified in real-life experiments for 3D parts of sizes ranging from 10 to 15 mm up to 40 mm.

## Introduction

Additive manufacturing (AM) is a rapidly growing technology that has the potential to revolutionize the way products are designed and manufactured. Predictive simulation plays a crucial role in AM as it allows for the assessment of the performance of the manufactured part before its actual production. Laser powder bed fusion (LPBF) stands as the most popular 3D printing method for creating functional metal parts, employing a laser to melt powder into the desired shape.

A significant challenge in metal part production through LPBF technology lies in the formation of residual stresses, which can lead to buckling and warpage. Residual stresses emerge from the relaxation of the crystal structure in metal alloys during significant plastic deformations. Even minor plastic deformations contribute to an increased dislocation density, which accumulates at the grain boundaries^1,2^. Moreover, uneven heating during the growth process causes substantial shape deformations. Experimentally adjusting the printing conditions to address this issue is a complex and costly procedure. It necessitates considering a wide array of parameters within the experimental setup, understanding the behavior of specific materials under thermal loads, and accounting for the influence of different metal powders. Consequently, numerical modeling of the 3D printing process emerges as the optimal solution for tackling these problems.

Simulation software packages can be utilized to assist in correcting shape deformation in metal alloy parts produced by LPBF. By numerically modeling the entire 3D printing process, which includes powder deposition, laser heating, cooling, and subsequent phase transitions, the software package can not only identify potential areas of macro-scale shape deformation and micro-scale defects but also suggest strategies to correct or eliminate them. In particular, the simulation can recommend adjustments to laser parameters and powder feed rate to attain the desired temperature distribution within the powder bed, thus enabling better control over its shape and reducing residual stresses, as well as addressing surface and volumetric defects such as inhomogeneity and undesired porosity in regions of partial melting. In general, simulation software for AM serves as a valuable tool for refining the design and production of 3D printed parts. However, it is essential to acknowledge the drawbacks associated with using this type of software, such as the cost, complexity, and accuracy of the results.

There is a significant leap ahead in the development of new and improved software packages for additive manufacturing. Large vendors are contributing to this research. For instance, AlphaSTAR^3^ is a powerful simulation product for additive manufacturing. It supplies a comprehensive suite of tools for simulating the entire AM process, from design to post-processing. It offers a wide range of features, including 3D printing simulation, material selection, and process optimization. ESPRIT Additive PBF^4^ is another great simulation product for additive manufacturing. It includes a library of materials and processes, allowing users to simulate their designs quickly and accurately. Oqton^5^ supplies a suite of tools for simulating the AM process. Whole, AlphaSTAR, ESPRIT Additive PBF, and Oqton are excellent simulation products for additive manufacturing. Additionally, they offer a range of advanced features, such as automated design optimization and support for multiple materials. Many of these packages utilize the finite element method (FEM)^6^ to solve all the necessary governing equations. This method is especially well-suited for predicting the behavior of materials with complex properties, while the finite difference method (FDM)^7^ and the finite volume method (FVM)^8^ are better suited for predicting the behavior of large-scale systems. Moreover, these methods can be combined and used together to optimize the AM process.

Although classical FEM/FVM-based approaches are relatively well-known, there has been a surge of recent efforts aimed at minimizing the runtime of part-scale models for the LPBF process. The majority of these works employ FEM-based solvers, incorporating specific modifications to establish correspondence between meso- and macro scales, enabling more efficient and accurate simulations.

Specifically, Moran et al*.*^9^ present a part-scale thermal modeling concept that rapidly predicts thermal defect distributions in additively manufactured components with high accuracy. Utilizing the principle of superposition, it simulates complete thermal histories without spatial or temporal coarsening within the timeframe of real builds. The simulations offer valuable insights into thermal defects, influenced by factors such as power schedule, component geometry, scanning strategy, and heat accumulation. In their subsequent work^10^ the authors present an improved superposition-based FEM approach that involves summing analytical solutions of temperature distribution in a semi-infinite medium derived from a moving laser source. The method utilizes adaptive mesh coarsening away from the zone of interest, but it is limited to a simplified linear heat problem. The proposed method can only compute the temperature field and lacks mechanical simulation, and it is restricted to linear heat equations, which makes it unsuitable for materials with spatially non-uniform temperature-dependent thermal transport properties, such as varying heat conductivity, density, and specific heat.

Gouge et al*.*^11^ conducted multi-scale modeling of fused material in representative volumes and the entire part to predict thermal history and distortions during the LPBF process. They utilized the well-known commercial AM simulation tool, Autodesk Inc. Netfabb Ultimate, which incorporates the Pan Solver, enabling multi-scale modeling with an adaptive resolution FE voxel grid that reduces the size of the matrix for more efficient solving. The heat conduction equation is treated as non-linear, while the mechanical structural solver considers elastic, plastic, and thermal strain, with plasticity computed using the von Mises yield criterion and the Prandtl-Reuss flow rule. Despite its superior accuracy compared to experiments, the solver requires a powerful PC to run efficiently, because it deals with 4D tensors. Specifically, using a 24 Core CPU and 192 GB RAM, a full LPBF simulation takes approximately 20 min to complete.

Another possibility for part-scale thermo-mechanical modeling of distortions is to utilize the sequent flash method proposed by Bayat^12^. This method involves an initial multi-scaling law to partition the numerical model (written in Python) and minimize computations at the meso-scale, while employing classical FEM at the macro-scale (part-scale) with the assistance of the Abaqus solver. The solver accounts for non-linear heat equations and material elastoplasticity, implemented through standard linear strain decomposition into thermal, elastic, and plastic strain tensors, with the latter computed using the J2 flow theory that uses a stress-space yield function to describe the onset of plastic deformation in a material. However, despite avoiding meso-scale computations, the method remains fundamentally FEM-based, resulting in a significant time requirement (up to 60 h) to achieve a 99% correspondence between simulated and measured deflections. In some cases, this duration exceeds the actual real-life printing time.

Summarizing all the listed FEM-based models and frameworks allows us to identify their shared advantages and drawbacks.

Pros: These models enable fast semi-analytical FEM solutions for heating, providing accurate volumetric strain–stress distributions of printed parts. They also account for the elastoplastic behavior of materials and support adaptive non-uniform meshes.

Cons: While the heat conductivity equation is computed quickly, the FEM’s need to construct and solve large matrices of linear equations, particularly when dealing with tensor variables like a stiffness tensor, demands significant computational effort and a substantial amount of RAM for structural mechanics solvers. This computational burden is especially pronounced when integrating meso-scale and part-scale levels of detail. Consequently, simulations can take up to half an hour to complete on supercomputers or even several decades of hours on mid-level computing platforms. Furthermore, although these frameworks can predict shape distortions after LPBF growth of a metal part, it seems they do not provide any specific recommendations to users on how to mitigate these undesired effects.

When dealing with complex materials or components that have experienced significant deformation or thermal history, another family of LPBF-related solvers are introduced based on an approach that is similar to classic FEM, but differs in a way how to apply thermal stresses on deposited layers of material. The method is called the “inherent strain method” (IS) and it is a computational technique to account for the effects of pre-existing or inherent strains in a material or structure. The IS involves modifying the initial state of the material with the known inherent strains before applying the external loads. By accounting for the pre-existing strains from already deposited and sintered powder layers, the simulation results more accurately reflect the actual behavior of the material deposited at current and consequent layers.

Liang et al*.*^13^ have developed a modified inherent strain method (MIS) for predicting residual deformation on the part-scale during the LPBF process. They employ a mean inherent strain vector applied layer-by-layer, effectively accumulating residual deformation and warpage. The model successfully reproduces all inelastic strains, including creep strain, arising in the part due to thermal load from the moving laser source. Small-scale mean inherent strain in a representative volume element is computed through classic FEM while solving the equation of zero divergence of the total stress tensor. The model has been successfully applied for layer-wise LPBF simulation, incorporating asymptotic homogenization and considering various laser scanning strategies^14^.

The same research group has incorporated the modified inherent strain method (MIS) into the Ansys Structural package^15^, creating an efficient multiscale LPBF process modeling framework to predict macroscale distortions. They have also considered the effective thermal conductivity of the powder bed. According to the authors, the simulation time can be significantly reduced from days to hours compared to single-scale FE modeling for each real-life deposited layer. The model’s performance was tested on a desktop PC with 8 CPU cores, demonstrating excellent agreement with experiments and superior computational efficiency when compared to conventional coupled thermal-structural pure-FE solvers. To achieve such a performance speed-up, two approaches are adopted. First, several real-life physical layers of deposited material are merged into a single-element layer activated at specific deposition times in a quasi-static analysis. The second reason for its efficiency is that coupled thermal-structural simulations are only performed in a representative volume at the micro-scale to extract inherent strains, which are then applied as thermal expansion coefficients at the macro-scale. Despite the listed advantages, the MIS-based simulation framework presented in that work has some limitations. The key bottleneck of the method is that the layer-by-layer simulation is highly nonlinear due to elastoplasticity, causing the simulation time to increase nonlinearly with the use of more equivalent layers. Additionally, the model requires mandatory calibration to ensure that the effective heat source and deposited material properties produce effects consistent with the real-world behavior.

Having shown great potential, the MIS was further improved by Dong et al.^22,28^ to enhance the accuracy of residual stress prediction, resulting in MIS_NEW. In contrast to previous approaches, the modified MIS incorporates an additional solution stage at each time step, utilizing thermomechanical properties at an elevated temperature computed from the inherent strain extraction step. This refinement significantly enhances the model’s capability to predict residual stress and distortions, even for small parts. By employing the proposed MIS_NEW for LPBF simulations, the authors achieved results that not only matched but even surpassed the accuracy of experimental measurements, outperforming commercial Ansys Additive Manufacturing (Ansys AM) framework. Consequently, the model emerges as a superior alternative to conventional FEM-based LPBF simulators. MIS seems not to have visible drawbacks; the only thing one should be careful about is that, due to the integration of micro- and macro-scale levels of detail, the model requires careful pre-calibration of its parameters to ensure accurate predictions.

In total, the above analysis highlights significant advancements in pure FEM-based, hybrid analytical, and MIS-based approaches. These methods have shown promising improvements in addressing various challenges associated with the LPBF process. However, it is important to note that solving the coupled thermal-stress problem using FEM/FVM/FDM and their variants remains a resource-intensive procedure. This is primarily due to the necessity of reassembling the global matrices of control systems containing linear algebraic equations at each time step and every instant of new powder layer addition. Therefore, while these approaches have seen notable progress, there is still room for further optimization and efficiency enhancement to minimize the computational difficulty in modeling the LPBF process, especially when considering nonlinearities, nonuniformity and anisotropy of materials. To solve this problem, we introduce a hybrid Lagrangian solver which combines stability with calculation speed and relative ease of implementation.

Lagrangian methods^16^ are powerful tools used for simulating the behavior of soft bodies, molecular dynamics, and solving particle motion equations. These methods rely on the principle of energy conservation, where the total energy of the system remains constant, enabling the calculation of particle motion from initial conditions. A significant advantage of Lagrangian methods is their relatively straightforward implementation, with the use of Euler–Lagrange equations to solve particle motion equations. Additionally, these methods offer high flexibility and can simulate a wide range of physical phenomena, including the behavior of flexible bodies such as cloth, jelly, and rubber, as well as rigid bodies like blocks, beams, and shells. Moreover, Lagrangian methods are efficient, making them well-suited for applications in computer graphics, where speed is critical.

The proposed approach for correcting shape deformation (CSD) involves using the classical finite difference method to solve one-dimensional heat conduction equations along thin beams connecting nodal points of the deformed mesh, enabling the modeling of volumetric heat propagation. The resulting temperature field at the nodal points serves as input data for the structural solver, based on replacing the continuous medium with a system of material points. Each point’s mass is equivalent to N/m_c_, where N is the number of particles in the voxel mesh cell, and m_c_ is the mass of this cell, assuming a uniform volume density of the material. These points are interconnected by virtual massless quasi-one-dimensional beams through which mechanical interactions propagate. By simulating the thermal distribution inside the part during its growth at the macro level, it becomes possible to identify zones of high temperature gradients, which directly contribute to thermal stresses. The obtained thermal map allows for the calculation of the final mechanical stresses and displacements experienced by the printed part under thermal load. This information is crucial for making further compensations by adjusting the initial shape of the printed part.

## Materials and methods

### Materials and experimental equipment for SLS printing, operating conditions

The 3D parts were printed at a 3D printer TruPrint 1000 (Trumpf, Germany) of stainless-steel powder (Höganäs AB 316L, Germany) with laser wavelength 1.07 μm. The analysis of the powder is presented for example here^17^. The print tasks were prepared in Materialise Magic software (ver. 25.0.2.435) and transferred by build preprocessor (Trumpf, ver. 3.0). The printing process parameters were chosen according to the manufacturer recommendations and shown at Table 1. Scan strategy “chess x–y, 4 mm” was used, the pattern shift was 2.7577 mm by X axis, and 3.3527 mm by Y axis. Laser scanning was by unconnected zig-zag route. The process was carried out without preheating either the powder or the base plate.

**Table 1:** LPBF process parameters.

Parameters	Value
Laser power	113 W/70 W (for contour)
Laser beam diameter	55 µm
Hatch spacing	80 µm/1 mm (for supports)
Layer thickness	20 µm
Laser scan speed	700 mm/s/500 mm/s (for contour)
Gas speed (Ar)	2.5 m/s
Oxygen level	< 0.3 at %,
Pressure in chamber	1 bar
Wavelength	1.07 µm

**Figure 1:**
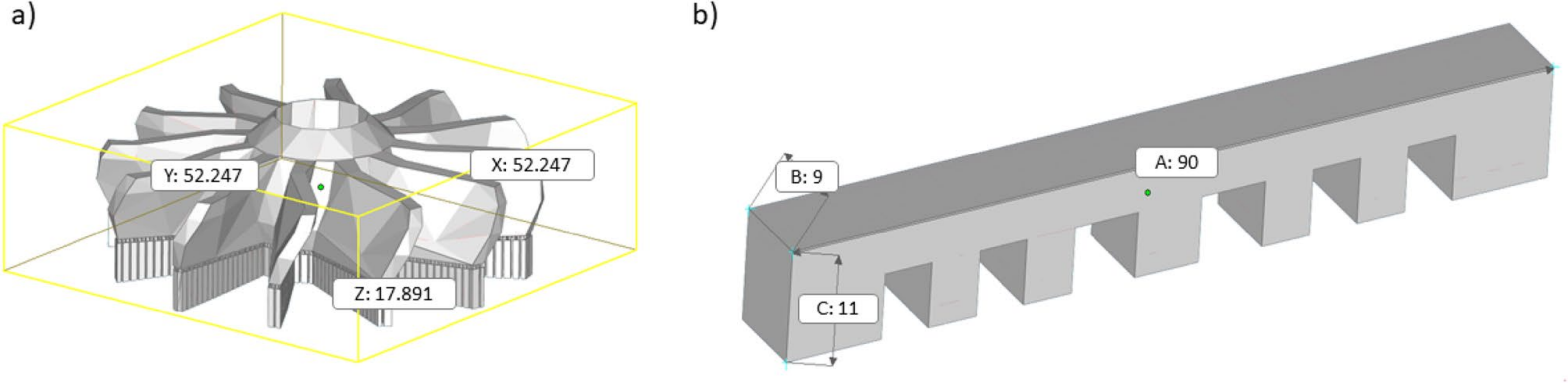
CAD appearance of printed 3D parts: (**a**)—turbine, (**b**)—comb/lattice. The geometric dimensions are indicated on the parts.

For working out the CSD procedure, the following samples of turbine and lattice prototypes were manufactured (Fig. 1a, b). For the “turbine” part (Fig. 1a), the “block” type supports were used, the “comb” (or “lattice”) part (Fig. 1b) was created without supports. The outer part of the supports had a section with a thickness of 500 μm, in the near the part the support section narrowed up to 300 μm. This solution improves the efficiency of the heat dissipation through support to the base of the platform. On the other hand, support with more thin sections is easier to separate from the manufactured part. The calculated volume of the turbine was 7199.646 mm^3^, the volume of supports was 948.77 mm^3^. For the comb part, which was printed without supports, the volume equaled 67,030.29 mm^3^.

**Figure 2:**
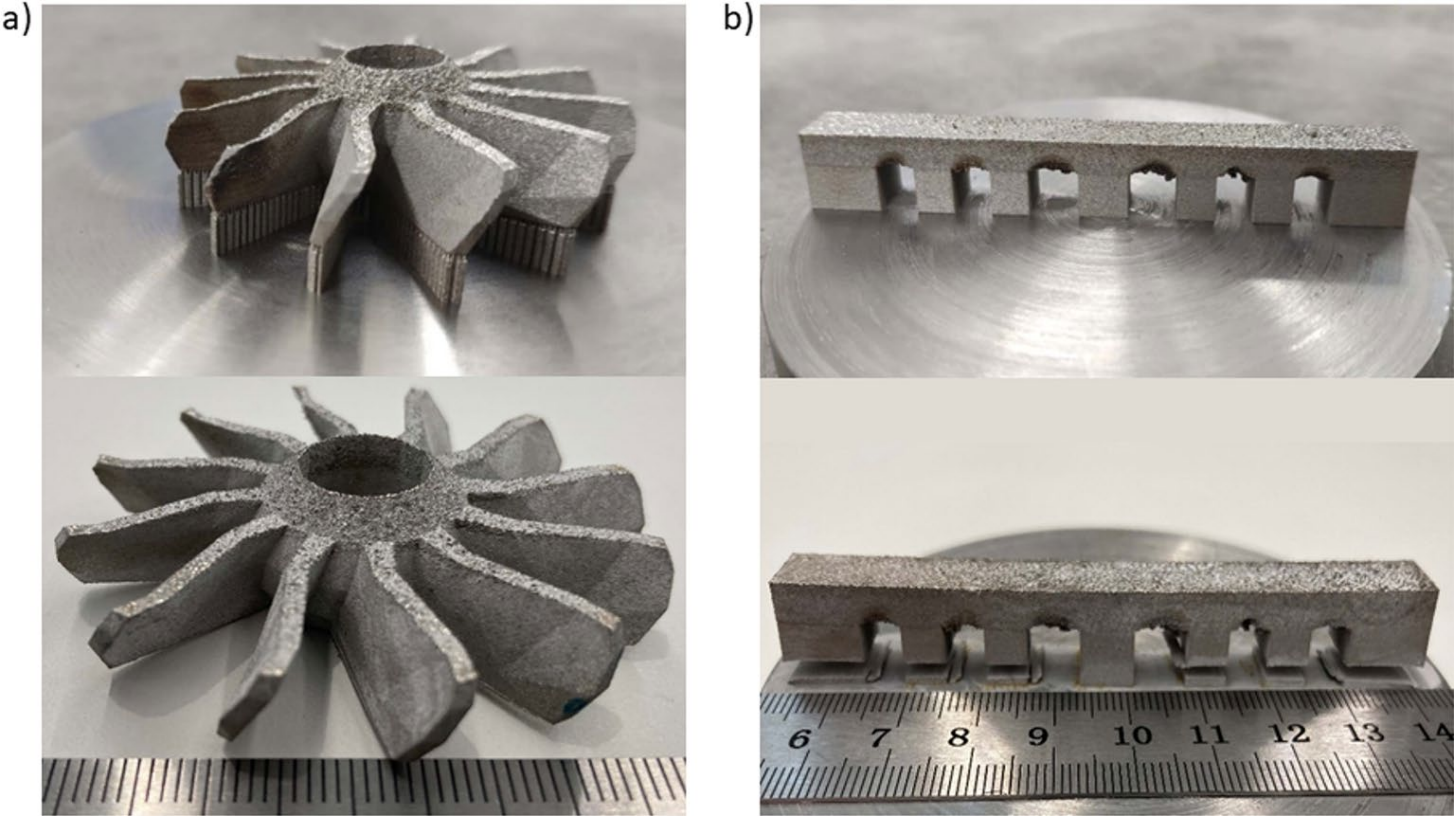
Appearance of printed 3D parts (**a**—turbine, **b**—comb/lattice) on substrate before (top) and after cutting off from substrate (lower row)**.**

Figure 2a,b presents 3D printed parts on substrate before (top) and after cutting off (bottom row). The arched effect between the teeth of the comb is clearly visible. The effect is essentially a distortion of the planar shape of the ceiling between the hexahedral teeth/legs/columns of the comb. As a result, the ceiling becomes arc-like with noticeable undesired spurs and grooves. This primarily occurs due to the burning and etching of powder material in the hanging zones of a printed part. The arching effect becomes even more pronounced as the distance between the combs’ teeth increases. The levelling time of each layer in both cases was 5 s. There were 550 layers in the production of a comb. Full time in the formation of each layer of comb—30 s. The separation from the platform led to the visible deformation of the “comb” product along the edges. The departure from the plate on the left was more than on the right.

During the production of the “turbine” part, grown right from the platform, 888 layers were needed, of which 200 layers form the supports, and the remaining 688 are responsible for the turbine itself. Full time to manufacturing of a layer during the construction of a turbine was 60 s. The presence of supports during “turbine” creating turned out favorable and minimized residual thermal stresses after a separating from the platform.

**Figure 3:**
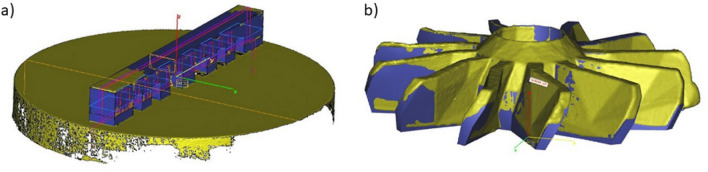
The overlay result of the 3D scan for printed details on the initial CAD sources.

3D scanning machine RangeVision Pro (MekSystems Oy, Finland) was use for surface digitalization of 3D printed samples. Scanning of the 3D printed product’s surfaces were carried out twice. The first time, the part surface was recorded on the construction platform. The second time, the procedure was repeated for the same part, after its separation from the platform. Moreover, in both cases, the parts itself were strictly centered by triangulation methods to avoid instrumental deviations associated with poor centering. The centering accuracy corresponded to the instrumental accuracy of 3D scanner and equal 18−1 m. ScanCenter software had built-in post-processing features to quickly align and merge made scans into a complete 3D model (STL format) without the external software. Figure 3a,b shows the color allocation in places of inconsistency of the initial STL files of the “lattice” (a) and “turbine” (b) parts with 3D scanned samples after 3D printing.

Material parameters, such as Young’s modulus and Poisson’s ratio, were obtained from results of tensile tests performed according to the ASTM E8/E8M procedure, using an Instron 5969 testing machine^18,19^.

### Governing equations and solution pipeline for thermal-structural model

#### A concept for a hybrid Lagrangian-finite-difference solver and a morphable mesh

The proposed solver is based on the concept of replacing the continuous matter with a framed model of material points (meta-particles) organized in a deformable voxel mesh and connected by virtual elastic rods of non-zero cross-section, along which the elastic force is applied and heat is propagated. Unlike Euler methods (FEM, FVM, FDM), the mesh is Lagrangian and therefore deformable, as at each time step the coordinates of its vertices (nodes) are updated. A program in C# was developed for modeling the AM of a product and compensation for warpage. The program's algorithm consists of the following key steps:

1. Import the reference geometry of the printed part surface in a stereolithography (STL) format.
2. Generate a volumetric mesh. The mesh cells are a uniform layer of cubes of the same size, which simplifies the task of slicing the mesh during calculations. Grid is partitioned into horizontal layers. Cell and vertex indexing correspond to the VTK format for convenient export of obtained results to that file format, to be opened in the ParaView scientific visualization software.
3. Converting of laser system parameters (scanning speed, spot diameter, power, absorption coefficient) into layer temperature during scanning.
4. A thermal-stress calculation of product warpage under a given operating mode.
5. Transferring the displacements calculated on the volume mesh to the STL surface mesh by associating the nearest vertices and later transformation. Thus, an STL file having the deformed geometry of the printed product is obtained.
6. Definition of a scaling coefficient and forming a compensating geometry. This allows an STL file to be obtained containing the geometry that, when printed under specific mode, will produce a part of the desired shape.

**Figure 4:**
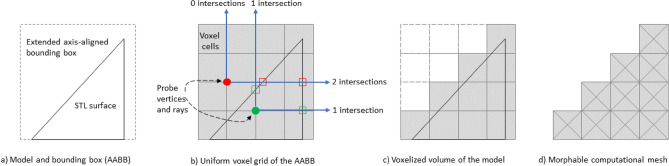
Morphable voxel mesh generation pipeline based on faceted surface mesh of the STL model. This schematic is in 2D although the actual mesh is 3D.

Since the reference geometry of the product is imported in STL format and contains information only about its surface for conduction of coupled thermal-strength calculation, a volumetric mesh (Fig. 4) representing cubic cells or voxels with 8 vertices each is required. To construct a voxel mesh enclosed within a given STL surface, the following steps are necessary:

1. Determine the enclosing volume, whether it takes the form of a hexahedral parallelepiped or an axis-aligned bounding box (AABB), and find the minimal and maximal x, y, and z coordinates among all the faces of the STL mesh (Fig. 4a);
2. Expand the obtained enclosing volume by 0.1 times in three directions, except for the negative direction along the vertical axis (to align the first layer of cells with the substrate level on which the part is grown). Then, generate a uniform finite element-like 3D mesh within the expanded volume (Fig. 4b), with a degree of discretization corresponding to the level of detailing present in the initial STL model. The purpose of extending the bounding box by 10% beyond the actual STL surface is primarily to ensure full coverage of all cells in the volumetric mesh that are near the surface. This extension prevents any instances where the cell volume barely overlaps with the STL surface, which could lead to cells being missed or not fully represented in the mesh. By providing this additional “buffer layer”, we guarantee that the mesh accurately captures the surface geometry without any gaps or omissions. Below, we present the details of the meshing algorithm.
3. Ensure the inclusion of 8 points for each hexahedral cell of the mesh inside the volume enclosed by given STL surface, as it represents the most accurate criterion, or alternatively, use only the cell’s centroid, which is a less accurate criterion. To assess the point’s inclusion within the closed surface, for each vertex inside the voxel mesh, three rays are casted towards “infinity” beyond the limits of the enclosing volume. If, during the process of casting these rays, at least one ray from the checked point (consider green dot in Fig. 4b) crosses the STL surface an odd number of times, then the point lies inside the surface. Conversely, if at least one ray from the checked point (represented by the red dot in Fig. 4b) does not cross the STL surface or crosses it an even number of times, then the point is located outside the surface. Determining whether the check ray intersects the STL surface involves solving a known problem of ray-triangle intersection in three-dimensional space. Due to the finite non-zero machine epsilon, which represents the precision of mathematical operations on floating-point numbers, certain cells located very close to the provided STL surface may pose challenges in determining whether they lie inside or outside the volume of the printed part. As mentioned in step 2, to address this issue, the bounding box containing all the cells to be checked is extended to generate several additional layers of cells. This extension helps eliminating the ambiguity and ensures more accurate inclusion determinations for cells that are in close proximity to the surface.
4. Finally, from the voxel grid filling the entire bounding box, only cells filling the volume of the STL surface remain (Fig. 4c). After that, all vertices inside each cell of this grid are connected pairwise with virtual links or rods of rectangular cross-section (Fig. 4d). Vertices found laterally and diagonally are connected to each other. Thus, each cell of the grid is a rigidly connected frame structure of tetrahedra and a cube surrounding them. Such a topology prevents the cell from folding into a flat figure, since it has triangles (that are fundamentally rigid figures) in its basis.

#### Heat conduction model

The primary task in laser melting modeling is to find the temperature of the material surface of the part induced by the absorption of radiation from the moving laser beam. Approximate solutions of the heat conduction equation obtained for instantaneous sources can be used for laser exposure with focused pulse. For a half-infinite space, there is a known formula for the surface temperature at the center of the moving laser beam^20^:

1 $$T=(1-R)\cdot \frac{P}{2\pi kr}\cdot \mathrm{exp}\left[-v\cdot r\frac{{c}_{p}}{2k\rho }\right]$$

Where T is the temperature [K], R is the coefficient of reflection of the laser radiation by the material, P is the laser power [W], *v* is the laser scanning speed [m/s], *r* is the radius of the laser spot [m], *k* is the coefficient of thermal conductivity of the material [W/(m*K)], ρ is the density of the material [kg/m^3^], and *c*_*p*_ is the specific heat [J/(kg*K)]. Laser scanning speed refers to the speed at which the laser beam moves across the powder bed during the LPBF process. However, the formula (1) does not consider the possible consumption of latent heat of phase transition when processing the material. To take this into account, intuitively, instead of use the full laser power *P*, it is necessary to use its reduced value (*P*—δ*P*), where P is the amount of energy delivered by the laser per unit of time and δP is the power absorbed due to the phase transition. From the dimensional theory it is clear that δ*P* = *L*_*m*_ * *m*/*τ* = [(J/kg)*(kg)/(s)] = [W], where *L*_*m*_ is the latent heat of melting, m is the mass of the melt (that is equal to ρ*S_b_*d, where *ρ* is the density of the material, *S*_*b*_ = π*r^2^ is the cross-sectional area of the laser beam, *d* is the depth of penetration of the radiation), *τ* = 2*v/*r* is the laser residence time on a metal surface when the spot is moving with linear velocity *v*.

**Figure 5:**
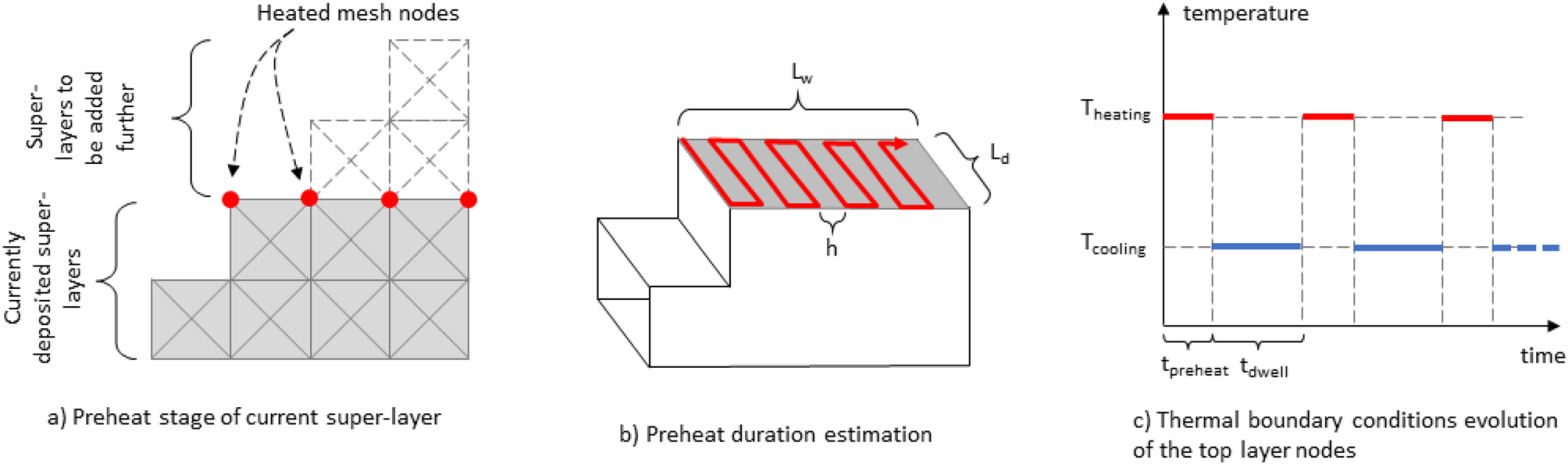
Schematic of thermal boundary conditions for super-layers of deformable mesh estimated based on approximate laser trajectory on deposited layer.

Once the temperature of the material layer in the laser heating zone is determined, this temperature is set as the boundary condition at all upper nodes of the deformed mesh of the current deposited powder layer (Fig. 5a). However, when printing products with dimensions up to ten centimeters, the number of powder layers may reach 1000, which significantly increases the number of mesh cells and calculation time. Therefore, we consider just “super-layers” in the mesh. The super-layer height coincides with the height of one mesh cell, and at the same time it can include up to several tens of real powder layers. The laser exposure time for the super-layer t_super_ is recalculated according to the irradiation time of a single real powder layer t_single_:

2 $${t}_{super}={t}_{single}\cdot {N}_{re\_sup}$$

where *N*_*re_sup*_ is the number of real powder layers in the super-layer of cells.

The irradiation time of the powder layer by the laser (or preheat time) depends on the surface area of the part layer, the laser scanning speed, and laser spot trajectory. Usually, this time is known for typical LPBF machines, for which a track-file with the location of the center of the laser beam at each moment in time is generated. However, the layer exposure time can still be estimated if it is assumed that the trajectory of the beam on the layer surface consists of a straight piecewise poly-line (Fig. 5b). If the diameter of the laser beam focus (h) and the surface areas of the applied layer (width *L*_w_ and depth *L*_d_) are known, the entire laser trajectory/path can be divided into L-shaped sections. The number of such sections will be equal to *N*_h_ = *L*_w_/*h*, and the length of each section will be *L* = *L*_w_/*N*_h_ + *L*_d_ = *h* + *L*_d_. Then it is easy to calculate the total path of the laser beam, which is then recalculated into the time of laser action on the layer:

3 $${t}_{preheat}=\frac{laser\, path\, length}{laser\, speed}=\frac{L\cdot {N}_{h}}{v}=\frac{(h+{L}_{d})\cdot {L}_{w}}{v\cdot h}$$

It should be noted that the preheat time, denoted as *t*_*preheat*_, may be equal to either *t*_*single*_ or *t*_*super*_, depending on the mesh resolution used. If the number of vertical layers in the mesh is equal to the total number of powder layers deposited in real life, then *t*_*preheat*_ equals *t*_*single*_. However, if the mesh has a lower resolution and cannot distinguish every individual powder layer, the layers are automatically combined into super-layers, and the laser residence time, while influencing them, is rescaled to *t*_*preheat*_ = *t*_*super*_. This approach ensures consistency in the energy applied per unit time per heated volume. The cooling time of the super layer *t*_*dwell*_ is calculated using the same method as the preheating *t*_*preheat*_, as described in expression (2). The only distinction is that, for cooling down, *t*_*single*_ corresponds to the average time taken for cooling and deposition of each of individual real-life powder layers, which together form the currently processed super layer.

After the super-layer is preheated for a time, *t*_*super*_ or *t*_*preheat*_, the boundary conditions on the top nodes of the current layer are replaced with the working chamber temperature (Fig. 5c), and the super-layer cooling time, *t*_*dwell*_, is calculated from the powder layer cooling time, according to Eq. (2).

After the temperature boundary conditions of the super-layer of the deforming mesh and the periods of their activation have been determined, the calculation of the non-stationary heat transfer from the top boundary of the applied (activated) super-layer to the depth of the printed part can be performed. For the deforming mesh, heat should propagate from node to node along the links between them. At the thermal calculation stage, the length of the links does not change, and the heat conduction equation is formulated in one-dimensional form, since it is solved on a thin rod:

4 $$\frac{\partial T(r,t)}{\partial t}=\frac{\partial }{\partial r}\left(a(r,t)\frac{\partial T(r,t)}{\partial r}\right)=a(t)\frac{{\partial }^{2}T(r,t)}{\partial {r}^{2}}$$

In this context, *r* represents the linear coordinate along the link, measured from one of its ends, while *t* corresponds to time. The thermal diffusivity coefficient, denoted by *a*, is defined as the ratio of heat conductivity *k* to the product of density *ρ* and specific heat capacity *c*_*p*_, i.e., *a* = *k*/(ρ*c_*p*_). When these thermal properties are spatially non-uniform and time-dependent, the heat conduction Eq. (4) becomes non-linear (as indicated at the center of formula (4)). However, if the heat diffusivity *a* remains uniform along the link's length, the equation simplifies to the Laplace equation (as shown on the right-hand side of formula (4)). The solver automatically checks the value of *a* at the link’s corners and determines which form of equation to solve. If the values of *a* at the corners are very close to each other, it employs the average thermal diffusivity of the link, calculated as the arithmetic mean along its length. Thus, the three-dimensional heat conduction problem is reduced to solving a set of one-dimensional equations defined on each link.

Equation (4) is solved using an implicit finite difference method by dividing each link into a finite number of equal parts thus forming a one-dimensional grid along the link direction:

5 $$\frac{{T}_{i}-{T}_{i}^{prev}}{\tau }=\frac{\left({a}_{i+1}-{a}_{i-1}\right)\cdot \left({T}_{i+1}-{T}_{i-1}\right)}{{h}^{2}}+{a}_{i}\left(\frac{{T}_{i+1}-2{T}_{i}+{T}_{i-1}}{{h}^{2}}\right)={a}_{i}\left(\frac{{T}_{i+1}-2{T}_{i}+{T}_{i-1}}{{h}^{2}}\right)$$

where *τ* is the time step [s], *h* is the spatial step [s], *T* is the temperature at the current time step [K], ${T}_{i}^{prev}$ is the temperature at the previous time step, and *i* denotes the node index of the one-dimensional grid along the link. By writing the recursive Eqs. (5) for each node of the one-dimensional grid, we obtain a system of linear equations which is solved using the Gauss–Seidel iterative method^21^. As mentioned earlier, the solver automatically determines the appropriate form of the discretized FD Eq. (5) to solve. If the coefficient *a* is uniform along the specific link/rod, it solves the very right-hand side of the equation. However, if *a* changes significantly along the length of the link, it solves the central part of Eq. (5), which includes an additional term accounting for the product of differences in *a* and temperature computed between the link’s inner segments.

For the existence of a unique solution of the equation at the initial time, Dirichlet-type boundary conditions are imposed at the edge nodes of each link (rod). It is important to note that at all subsequent time steps, Neumann-type conditions of perfect thermal insulation are applied in order to allow for temperature change at the rod's ends. To smooth out the temporal solution, relaxation factor f = [0 … 1] is used, displaying the relative contribution of the predicted temperature value at the given time step to the resulting solution:

6 $$T=\left(1-f\right)\cdot {T}^{prev}+f\cdot {T}^{current}$$

After solving the heat conduction equations, the temperatures of all nodes in the three-dimensional deformable computational mesh are updated. This process is repeated until the relative difference of temperatures between two iterations in the entire mesh is less than the specified precision of 10^–4^, which is quite enough for practical purposes. The relative difference can be estimated using expression (28), with the value of L (length of a link/rod) being replaced by T (temperature).

#### Elastoplastic structural model

When replacing a continuous medium representing a stack of powder layers with a lattice of linked/bonded meta-particles (i.e. nodes of the deformable mesh) the distributed mass of the body must be concentrated. In this case, the mass of each meta-particle is equal to:

7 $${M}_{d}=\frac{{M}_{c}}{N}$$

where *N* is the number of particles in a voxel grid cell, and *M*_*c*_ is the mass of the solid material contained within that cell.

To transfer the elastic properties of a continuous medium to a frame model, it is necessary to also modify the Young’s modulus of these thin rods. The effective Young's modulus of the links should be equal to the ratio of the cross-sectional areas of the solid block and the rods of its frame. Only then, when the same force is applied, the displacements of the solid and frame models coincide. This follows from the formula of elasticity force for a one-dimensional rod or the Hooke’s law pointing that the force *F* needed to extend or compress a rod by some distance scales linearly with respect to that distance.

**Figure 6:**
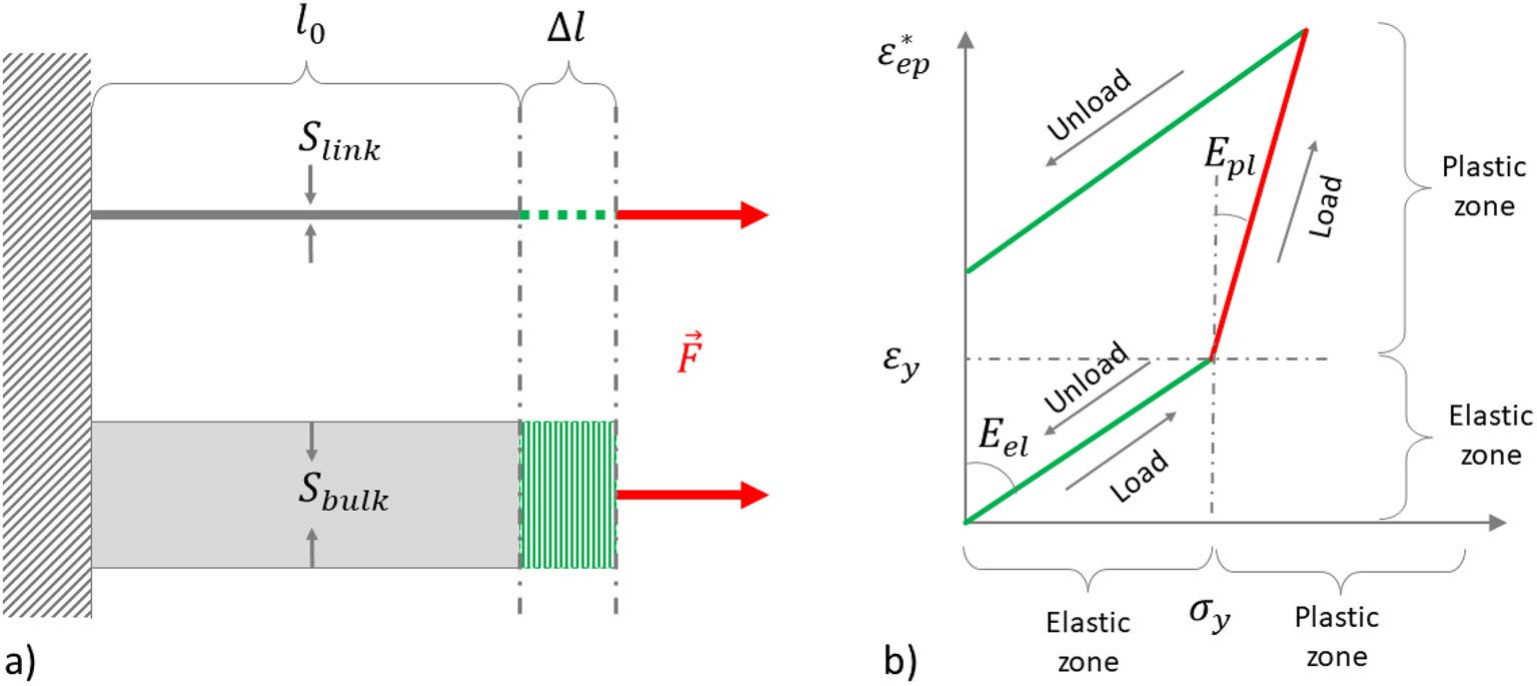
(**a**) Thin and thick rods under the same force applied to derive effective Young’s modulus of thin rod/link; (**b**) Schematic of bilinear elastoplastic behavior of material.

In further discussing the algorithm to compute thermal-load-induced stresses and convert them into deformations of each link connecting each pair of meta-particles, we have prepared a simplified schematic of the deformation of ideal thin and thick rods/links to compute effective structural properties of the material (Fig. 6a) and the way we integrate the stresses along the link (Fig. 6b). Note that the conventional order of “stress(*σ*)-versus-strain(*ε*)” on the simplified load curve has been swapped (Fig. 6b), because we derive 1D strains along the link from the given stress, which is estimated according to the thermal load. The curve represents a phenomenon of bilinear isotropic hardening, wherein the effective Young’s modulus of the link material changes depending on whether the external thermal stress increases or decreases in time. This curve is “simplified” for the sake of clarity and stands for the “strain-vs-stress” curve in an imaginary case wherein during a single time step of simulation, the thermal load changes significantly, but the elastoplastic physical properties of the material remain temperature-independent. That is why the curve is mostly linear, although during simulations it can be smoother, as we consider the thermal dependence of material properties. The load curve may also be non-linear because we integrate the strains in time, rather than following the pre-defined “strain-vs-stress” curve according to (23) and (25), where elastic (*E*_*el*_) and elastoplastic (*E*_*pl*_) Young’s moduli depend on temperature and thus changes over time, as will be described further in this chapter.

The elasticity force can be expressed from Hooke’s law through relative displacements, as well as from the stress tensor, which has the dimension of pressure—that is, a force per unit area of the rod’s cross-section:

8 $$F=k\cdot \Delta L= \sigma S$$

where Δ*L* is the absolute change in rod’s length or its displacement from the position where it was being unloaded; *k* is the stiffness coefficient; *σ* is the stress tensor considering just its principal component having the same direction as a force *F* along the rod; and *S* is the cross-section of the rod. Note that here and in the following discussion, we will not use tensor notation to describe the stresses, as we are dealing with one-dimensional rods where the stress is represented by a vector proportional to applied force F. Also, for a one-dimensional link/rod, the relative deformation is the same as the strain. Indeed, as the strain refers to the measure of the deformation of a material due to an applied force or stress, for a 1D rod, the deformation only occurs along one direction, and thus the strain can be calculated by dividing the change in length of the rod (i.e., the deformation) by its original length. Stresses, in turn, are related to deformations through the Young’s modulus:

9 $$\sigma =\varepsilon E=\frac{\Delta L}{{L}_{0}}E$$

where *ε* is the principal component of the strain tensor along the rod’s length or its relative elongation; *E* is the Young’s modulus of the rod’s material; Δ*L* is the absolute change in the rod’s length; *L*_0_ is the original length of the rod with no force applied.

Comparing these two relationships, we obtain the correspondence between the rod’s stiffness with its original length, the Young’s modulus of the material, and its cross-sectional area:

10 $$k=\frac{ES}{{L}_{0}}$$

According to Hooke’s Law, to ensure the elongations of two rods of different cross-sections to be equal when the same force is applied to them, their stiffness coefficients must also be equal. For thin (quasi−1D link) and thick (3D bulk) rods in Fig. 6a, these relationships can be written as:

11 $${k}_{link}=\frac{{E}_{link}{S}_{link}}{{l}_{0}}\equiv {k}_{bulk}=\frac{{E}_{bulk}{S}_{bulk}}{{l}_{0}}$$

It follows that in order to have the same deformation of a voxel cell of a continuous medium (bulk) and its frame model of thin rods (link) under the same applied external force, an effective Young’s modulus is used, which is equal to the ratio of the cross-sectional areas of the solid cell and the rods of its lattice mesh:

12 $${{E}_{link}=E}_{eff}={E}_{bulk}\cdot \left(\frac{{S}_{bulk}}{{S}_{link}}\right)$$

where *E*_*bulk*_ is the Young’s modulus of material [Pa]; *S*_*bulk*_ is the cross-sectional area of the cell mesh [m^2^], and *S*_*link*_ is the cross-sectional area of the link.

When elongation or compressing such links, the cross-sectional area of the link (*S*_*link*_) is not constant over time and is found by the Poisson’s ratio of the material:

13 $$\nu = \frac{{\left( {{\raise0.7ex\hbox{${\Delta h}$} \!\mathord{\left/ {\vphantom {{\Delta h} {h_{0} }}}\right.\kern-0pt} \!\lower0.7ex\hbox{${h_{0} }$}}} \right)}}{{\left( {{\raise0.7ex\hbox{${\Delta L}$} \!\mathord{\left/ {\vphantom {{\Delta L} {L_{0} }}}\right.\kern-0pt} \!\lower0.7ex\hbox{${L_{0} }$}}} \right)}}$$

where ∆*L* is the absolute increase in length of the thin rod (m), *L*_*0*_ is the initial length of the thin rod [m], *∆h* is the increase in thickness of the thin rod when it is elongated [m] and *h*_*0*_ is the initial thickness of the thin rod [m].

Using the expression (13), let us define the increment of the link thickness given the known increment of its length:

14 $$\Delta h = - \nu \left( {{\raise0.7ex\hbox{${h_{0} }$} \!\mathord{\left/ {\vphantom {{h_{0} } {L_{0} }}}\right.\kern-0pt} \!\lower0.7ex\hbox{${L_{0} }$}}} \right)\Delta L$$

Then the cross-section area equals:

15 $$S_{link} = h^{2} = \left( {h_{0} + \Delta h} \right)^{2}$$

Note that the expression (12) calculates the effective Young’s modulus of a quasi−1D rod, taking into account bare cross-sectional areas of both thin and thick rods, regardless of their actual shapes, which may be rectangular, circular, or even triangular, with appropriate area formulas. However, when dealing with the rod’s thickness, directly linked to the material’s Poisson’s ratio and absolute elongation, it is convenient to consider the rod to have a rectangular cross-section due to its simple expression (15) for computing the area, avoiding the use of irrational numbers.

Once the expressions to compute all the necessary parameters of the deforming link or rod have been defined, we can proceed with an explanation of the algorithm to compute coupled thermal-structural dynamics of a system of these rods. These rods connect the vertices of the deforming volume mesh, which fills the volume of a closed surface of the part to be printed. The algorithm for solving the structural problem in the developed solver consists of two stages:

1) Calculation of the expected (“desired”) length of each link/rod due to its pre-computed thermal expansion or shrinkage. At this stage, the edges of the links are not moving in space, but only the current length of the rod is predicted. The linear Hooke’s law is used to express the length of the link through strain:

16 $${\varepsilon }_{ep}^{*}=\frac{{L}_{current}-{L}_{0}}{{L}_{0}}=\frac{\Delta L}{{L}_{0}} \to {L}_{desired}={L}_{0}+\Delta L={L}_{0}+{L}_{0}{\varepsilon }_{ep}^{*}$$

where *L*_*desired*_ is the expected length of the link under thermal load; *∆L* is the elongation of the link caused by thermal expansion, following from the definition of the strain rate along a one-dimensional link; *L*_*0*_ is the initial length of the undeformed link under zero external load; ${\varepsilon }_{ep}^{*}$ is the elastoplastic relative deformations in the quasi-linear approximation.

2) Relaxation of links, in which the particles it connects are shifted along it until the length of each link is equal to the expected *L*_*desired*_ calculated at the first stage in quasi-linear approximation.

Algorithm for calculating relative deformations in a quasi-linear approximation for the first stage of the structural solver is the following:

Estimate the strain that is related to change of the rod’s/link’s temperature:

17 $${\varepsilon }_{th}=\alpha \left(T-{T}_{ref}\right)$$

where *α* is the coefficient of thermal expansion [K^−1^]; *T* is the average temperature of a link, calculated as the half of the sum of the temperatures of its corner points; *T*_*ref*_ is the temperature of zero thermal strain of the material.

2) Compute the strain rate of the link caused by an external thermal load:

18 $$\frac{d{\varepsilon }_{th}}{dt}=\frac{\left({\varepsilon }_{th}-{\varepsilon }_{th}^{prev}\right)}{dt}$$

where *dt* is the time step; *ε*_*th*_ is the relative thermal strain; ${\varepsilon }_{th}^{prev}$ is the relative thermal strain from the previous time step. This expression is necessary to determine the mode of external thermal loading (Fig. 6b):

19 $$\begin{array}{c}Load=\{1\, if \frac{d{\varepsilon }_{th}}{dt}\ge 0; 0\, othewise\} \\ Unload=\{1\, if \frac{d{\varepsilon }_{th}}{dt}<0; 0\, otherwise\}\end{array}$$

3) The mode of internal deformation of the link’s material (elastic/plastic) is determined according to the following criterion, when the external thermal load is less or greater than the yield one, that depends on temperature:

20 $$\left\{\begin{array}{c}\left|{\varepsilon }_{th}\right|<{\varepsilon }_{y}: elastic=1, plastic=0 \\ \left|{\varepsilon }_{th}\right|\ge {\varepsilon }_{y}: elastic=0, plastic=1\end{array}\right.$$

in which *ε*_*th*_ is calculated by formula (10), and the yield limit of elastic deformation *ε*_*y*_ is calculated as:

21 $${\varepsilon }_{y}=\frac{{\sigma }_{y}}{{E}_{el}}$$

where *σ*_*y*_ is the average yield strength [Pa], calculated as half the sum of the yield strengths of the points forming the link; *E*_*el*_ is the average effective Young’s modulus of the link points calculated according to formula 12.

4) Deformation is converted into quasi-elastic (linear) thermal stresses along the link according to Hooke’s law:

22 $$\frac{d\sigma }{dt}=\frac{d{\varepsilon }_{th}}{dt}{E}_{el}$$

where $\frac{d{\varepsilon }_{th}}{dt}$ is calculated by formula (18) and $\frac{d\sigma }{dt}$ is the increment of stress along the link due to thermal loading in the linear approximation. Quasi-elasticity implies a linear relationship between the stress tensor *σ* and the strain *ε*_*th*_ in the case of an elastic material with Young’s modulus *E*_*el*_: since $\sigma ={\varepsilon }_{th}{E}_{el}$, then their increments in time in the approximation of a slowly changing Young’s modulus will be equal: $\frac{d\sigma }{dt}=\frac{d{\varepsilon }_{th}}{dt}{E}_{el}+\frac{d{E}_{el}}{dt}{\varepsilon }_{th}\approx \frac{d{\varepsilon }_{th}}{dt}{E}_{el}$. It should be noted that, unlike the classical Hooke’s law for the static structural analysis of a continuous medium, (22) considers the strain, which is necessary to consider the effect of thermomechanical hysteresis that causes residual deformations.

5) The local slope of the “stress–strain” curve is found, which has the physical meaning of the “elastoplastic” Young’s modulus, which can be used as a proportionality coefficient between stresses and deformations:

23 $$\frac{1}{{E}_{slope}}=\frac{1}{{E}_{el}}\cdot elastic+\left(\frac{Load}{{E}_{pl}}+\frac{Unload}{{E}_{el}}\right)\cdot plastic$$

where *Load* and *Unload* are calculated according to formula (19), and *elastic* and *plastic*—according to formula (20), *E*_*pl*_ is the effective Young’s modulus of the link in the zone of plastic deformations, calculated as the average plastic Young’s modulus of its ends specified by the user.

6) The time derivative of the total deformation speed (in this formula *E*_*slope*_ changes over time) is being reconstructed:

24 $$\frac{d{\varepsilon }_{ep}^{*}}{dt}=\frac{d\sigma }{dt}\frac{1}{{E}_{slope}}$$

7) Quasi-elastic relative deformation is explicitly integrated over time:

25 $${\varepsilon }_{ep}^{*}={\varepsilon }_{ep}^{*,prev}+\frac{d{\varepsilon }_{ep}^{*}}{dt}dt$$

where ${\varepsilon }_{ep}^{*,prev}$ is the value at the previous time step.

Such an approach to time-integration of strain allows for the consideration of the elastic–plastic hysteresis on the loading curve in the “strain–stress” coordinates (Fig. 6b). The presented algorithm automatically considers cyclic external thermal loads, as it is differential in time (refer to expressions (18) and (22)–(25)). The thermal load along the link is converted into 1D strain (17). The change in strain during the time step is then calculated using the finite difference formula (18). Assuming the thermal stress changes slowly, the strain rate is converted into the rate of change of stress considering the temperature-induced elongations of the link are in elastic mode (22). The material elastoplastic behavior then becomes dependent on the applied linearized thermal stress. If the stress increases over time and its value is greater than the yield stress (represented by the red line patch on Fig. 6b), the material Young's modulus corresponds to the plastic zone, thus the slope of the “strain-vs-stress” curve is the reciprocal of *E*_*pl*_. In contrast, when a link cools down, the thermal load decreases (as indicated by the green line patch on Fig. 6b and expression (19)), but the curve slope is dependent on whether the material is in plastic or elastic mode, so the final slope is a combination of elastic and plastic Young's moduli, which are temperature-dependent. Knowing, the effective *E*_*slope*_, the pseudo-elastic first derivative of the thermal stress with respect to time is converted back to the strain rate (24), which is then integrated (25) in time using an explicit Euler’s scheme to reconstruct the expected change in strain along the link, which is its relative deformation. The “strain–stress” loading curve obtained via the expressions (17)–(25) may differ from the simplified one illustrated in Fig. 6b, as the material-dependent variables of Young’s modulus in the elastic and plastic zones depend on temperature and thus vary over time when integrating the strain. Thus, although the linear relations between strain rate and stress rate suggest that the slopes are linear, they (slopes) may change over time, resulting in a non-linear “strain–stress” load curve. This allows the elastoplastic model to work regardless of whether the laser-heated super-layer of powder layers is being applied for the first time or is preheated by subsequent layers. And newly fused powder layers can affect the residual deformations of previously applied layers when they are repeatedly heated and cooled by the surrounding layers. In all the test presented, the material’s yield strength is considered to be constant. However, the solver easily allows this property to be temperature dependent using a piecewise linear interpolated table, which consequently makes it implicitly dependent on the load history.

The second stage of the algorithm is pairwise relaxation of the distances between the connected particles of each link to achieve the elongations calculated in the first stage of the links. For each link, its corrective length is calculated as the difference between its current length and the required length *L*_*desired*_ calculated in the first stage from the thermal load according to (16–25):

26 $${\overrightarrow{r}}_{corr}=\frac{1}{2}\cdot {\overrightarrow{d}}_{12}\cdot \Delta L, \Delta L=L-{L}_{desired}$$

where ${\overrightarrow{d}}_{12}$ is the unit vector along the link from its first to its second point, *L* is the current distance between the ends of the link, *∆L* is the increment in the length of the link, and the factor 1/2 considers that the particles at the ends of the link move symmetrically away from its center by half the length of the link itself.

To achieve the desired length *L*_*desired*_, the endpoints of the link (the centers of which contain the connected material points) are iteratively shifted in opposite directions along the link direction vector:

27 $${p}_{i}^{(i+1) }={p}_{i}^{(i) }\cdot {\overrightarrow{d}}_{12}\pm {\overrightarrow{r}}_{corr}\cdot {\mu }_{p}, {\mu }_{p}=\frac{\langle D\rangle }{{D}_{p}}$$

at each iteration *i*, the mobility of the p-th point *μ*_*p*_ is inversely proportional to its diameter *D*_*p*_, which physically means that small and light points have high mobility and large points have low mobility due to the effect of inertia, where 〈*D*〉 is the mean diameter of the material point of the link (averaged over its two ends). The sign ± in (27) means that the first point moves in the positive direction along the link, and the second moves in the opposite direction. The iterations stop when the maximum relative length increment of all links in the deformed mesh does not exceed a given minimum number:

28 $$max\left|\frac{{L}^{(i+1)}-{L}^{(i)}}{0.5\cdot \left({L}^{(i+1)}+{L}^{(i)}\right)}\right|\le \varepsilon$$

And that is all about the thermal-structural solution pipeline on the deformed mesh of linked particles.

## Shape compensation algorithm

**Figure 7:**
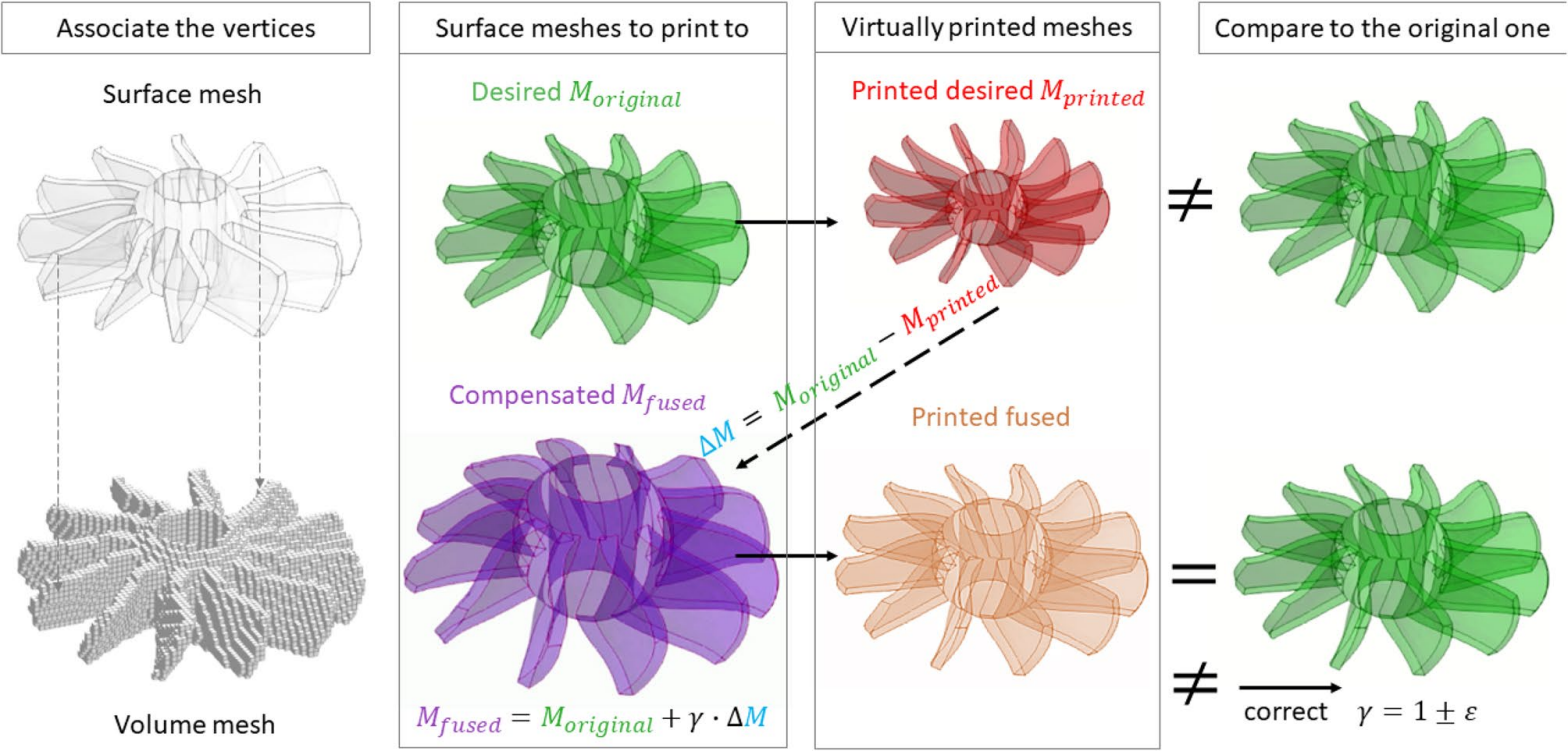
A schematic of the shape distortion compensation algorithm.

Once the thermal-deformation history of a virtually printed part has been obtained, it is necessary to implement its shape compensation algorithm to make the shape and size of the “printed” model perfectly match its desired “original” shape. Because of a 3D deformable mesh has been built from the voxelization of a surface mesh from an STL file, we need to associate the vertices of the surface mesh to the closest nodes of the morphable volume mesh before the simulation starts (Fig. 7, left). This procedure of vertex association is essentially a loop over all vertices of both surface and volume meshes to find those that are located within a given distance threshold of each other. If more than one vertex of a deformable volume mesh is close to a particular vertex of the surface mesh, then we take the closest one. Thus, before the solution begins, the STL vertices are associated with the nearest surface cells of the original voxel grid and the centers of these cells are remembered. After calculation of thermal-load-induced displacements, the displacement vectors of the centers of the surface cells of 3D deformed mesh are determined and added to the coordinates of the original STL vertices.

Once we know which nodes of the deformable volumetric mesh are close to particular vertices of the surface mesh, we can morph the surface mesh according to the deformable volume mesh after it has been virtually printed during the thermal-structural simulation. Keys steps of the mesh shape compensation algorithm are briefly illustrated in Fig. 7. Let’s denote the vector of vertex coordinates of virtually printed surface mesh as *M*_*printed*_. Then we can estimate the discrepancy (so called delta-mesh) of given original surface mesh loaded from STL file (*M*_*original*_ shown in green color in Fig. 7) from the printed one as follows:

29 $$\Delta M={M}_{original}-{M}_{printed}$$

The idea is to determine the compensated surface mesh (*M*_*fused*_ shown in purple in Fig. 7) fused from the original one and delta-mesh such that, once printed, it will perfectly match the original one, because its residual deformations due to printing will be compensated by the initial shape of the fused mesh:

30 $${M}_{fused}={M}_{original}+\gamma \cdot \Delta M$$

For example, if the printed mesh is smaller than the original one due to thermal shrinkage, then the compensated mesh will be larger and its shrinkage when printing will compress it to the size of the original mesh. The multiplier *γ* in (30) is a corrective factor that determines the contribution of the deviation (printed from the original) and the original geometry to the compensated geometry. It is close to one with small possible deviations in either direction. The corrective factor is dependent on the printing machine and its operating conditions. This factor may be corrected if printed “fused” mesh does not match the desired one fully. In our tests, the small parameter ε, which stands for the deviation of *γ* from 1, does not exceed 0.1. A positive value of ε is related to fast printing, resulting from high heating–cooling rates. Conversely, during relatively slow printing speed, when the part experiences lower temperature gradients, ε takes on a negative value. The exact value of ε is determined by performing a series of simulations for 3D printing of both the original and compensated geometries, ensuring that the virtually printed corrected geometry closely matches the desired original geometry with minimal deviation.

Now let us see the thermal-structural solution and distortion compensation algorithms on real cases.

## Sanity checks and performance tests

Here, we have prepared a set of synthetic tests to prove the basic functionality of the developed model and then to prove it in close-to-real-life conditions with subsequent comparison to experiments.

### Elastoplastic deformation of a rod due to predefined thermal stress

At the first stage to warm-up the solver it is necessary to make sure that the model correctly reproduces the residual deformations after thermal loading. First, the deformation of a part with a simple geometric shape (parallelepiped) is modeled under a given, uniform in volume, thermal load, the mechanical properties of which are presented in the Table 2. The properties of that test synthetic material are defined that way to make residual deformation more distinguish after the cool-down stage. This material is mostly similar to stainless steel in terms of temperature-independent properties, and its Young’s modulus in a plastic zone is two times lower than in the elastic zone before reaching the yield strength.

**Table 2:** Material properties of 1D rod under spatially uniform thermal load.

Property	Value
Young’s Modulus	2.0e + 11 [Pa]
Poisson’s ratio	0.3
Coefficient of thermal expansion	0.0001 [K^−1^]
Reference temperature of zero thermal expansion	300 [K]
Yield strength	9e + 09 [Pa]
Young’s modulus in the plastic zone	1.0e + 11 [Pa]
Density	8000 [kg/m^3^]

This is a test material with such properties that the residual deformations of the rod are visually well noticeable, and it is possible to compare the calculations with Ansys in a visible way. The physical properties of this material differ from those of the real material (Stainless Steel AISI 316L) listed in the Table 3, as one can notice.

**Table 3:** Temperature-dependent properties of AISI 316L stainless steel.

Property	Temperature range [K]	Value range
Density	300–3000	7954–5930 [kg/m^3^]
Coefficient of thermal expansion	273–1300	(1.457–1.945) * 10^–5^ [K^−1^]
Melting temperature	Any	1643 [K]
Young’s modulus	293–1473	(19.5–5.0) * 10^10^ [Pa]
Yield strength	300–1420	(22.5–1.5) * 10^7^ [Pa]
Tangent Young’s modulus in the plastic zone (when the stress exceeds the yield strength)	300–1420	(20–2.6) * 10^8^ [Pa]
Thermal conductivity	273–1700	12.97–27.24 [W/(m*K)]
Specific heat	300–1800	498–769 [J/(kg*K)]
Reflectance at the λ = 1.07 µm	Any	0.25

**Figure 8:**
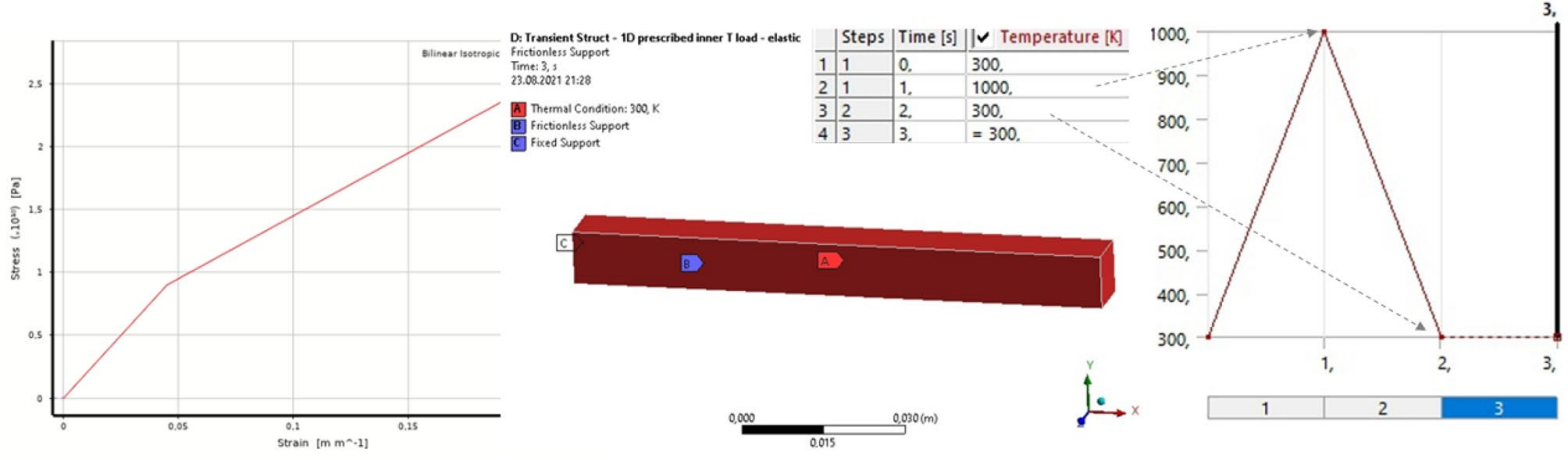
Ansys settings for pseudo−1D model of a rod. From left to right: “Bilinear isotropic hardening” material; modelled geometry and the boundary conditions; prescribed uniform thermal load vs time.

The parallelepiped is modeled as a quasi-one-dimensional rod with dimensions 0.01 × 0.1 × 0.01 [m]. It is composed of a set of four elastoplastic links along its length. To achieve similar conditions in Ansys Structural, the material is specified using the “Bilinear isotropic hardening” model, which corresponds to the data in Table 2. Throughout this study, we utilize the Ansys 19.2 Academic version. The thermal load is manually applied to the part, while Frictionless contact is applied to all four side faces to prevent the part from expanding. This setup allows for the prohibition of displacements normal to the surface, while still enabling the part to slide along. No thermal calculation is performed, instead in the “Transient Structural” block a pre-defined change in T is specified within the part as a function of time (Fig. 8, central and right parts).

**Figure 9:**
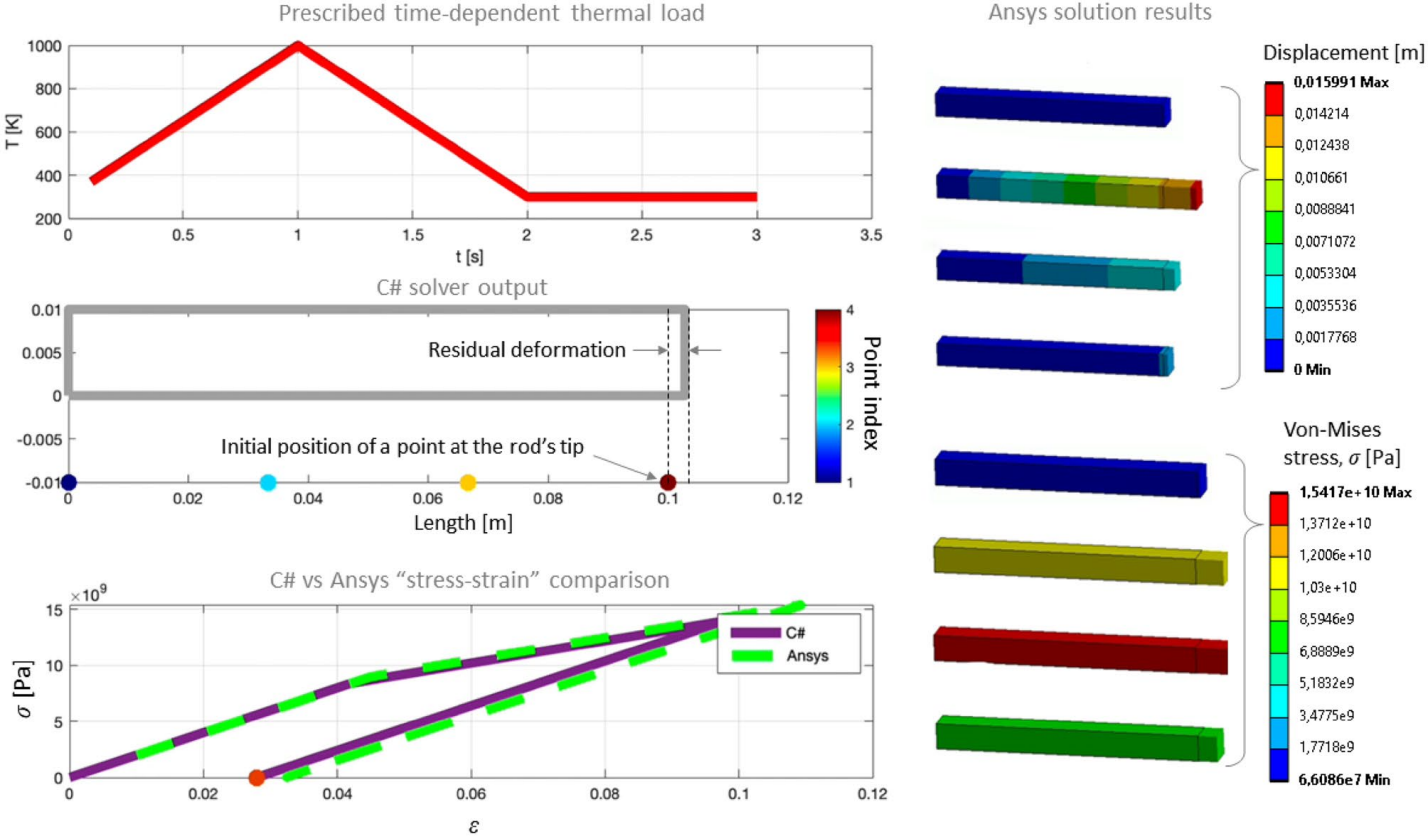
Residual deformations and the stress–strain hysteresis curve for a model of 1D rod under prescribed uniform thermal load. Left: custom C# solver. Right: Ansys Transient Structural analysis from top to the bottom: at 0 s, 0.95 s, 1.5 s and 2.9 s, respectively.

The calculations were performed using a custom solver written in C# and the Ansys Transient Structural solver, which is based on the Finite Element Method. At each time step, the strain and stress values at the free end of the rod were recorded to reconstruct the loading curve. The results show that both the custom and Ansys reference solvers accurately reproduce the strain–stress hysteresis curve, and the part exhibits residual deformations even after the external thermal load is completely removed at 3 s. (Fig. 9, left).

This is consistent with the expected behavior of a material with a bilinear stress–strain relationship. Indeed, the residual deformations are due to the change in the Young's modulus of the material with increasing and decreasing external (thermal) load (as shown in the Figs. 6b and 8). It is also noticeable that the rod deformations (shown at the top right in the Fig. 9, right) are visually proportional to the temperature loading curve (shown in red at the top left).

Although the deforming mesh of the custom solver consisted of only 4 points and 3 connecting links (Fig. 9, middle left), and the Ansys grid was a finite element static grid, the calculation results were close to each other. In particular, the residual deformations of the one-dimensional rod are: Ansys = 0.033 [m], C# = 0.028 [m], and the maximum stresses in the plastic zone are: Ansys = 1.542e10 [Pa], C# = 1.459e10 [Pa]. This proves that the custom solver is capable of reproducing both elastic and plastic deformations of the material. Therefore, one can proceed to more complex tests involving coupled thermal-structural calculations on the deformed Lagrangian mesh.

### Deformation of printed lattice-shaped part before and after the base removal

The next test is about simulation of 3D printing of a “comb” or lattice geometry described in Sect. 1 of “Materials and methods” chapter of this paper. In contrast to the first test of 1D rod, this geometry is modelled on full 3D deforming mesh having a real-life material with temperature-dependent physical properties. As usual, for welded applications one should avoid higher carbon alloys. That is why the material used for printing is AISI 316L stainless steel which basically is the low carbon (“L” stands for “low”) version of AISI 316 (0.08%C) which may be susceptible to intergranular corrosion in certain corrosive media. Its properties are given in the Ansys material database and other open sources and^12,18,19,23–26,28,29^. We have collected them in Table 3 providing corresponding temperature range to interpolate tabular data within it. These properties are used in our thermal and structural solver on deforming Lagrangian mesh implemented in C#. Physical properties of every elastoplastic link in a mesh are basically an arithmetic mean of these properties at the link’s corner points and may change during the simulation according to temperature values calculated at these points.

The reflection coefficient of 316L stainless steel at a wavelength of 1.07 µm (that is typical for our laser) depends on several factors, such as the angle of incidence and powder surface roughness. However, in general, stainless steel is known to have a relatively low reflectivity in the visible and near-infrared spectrum. According to public sources^26,30^, the average reflectance of 316L stainless steel at a wavelength of 1.06 µm (which is close to 1.07 µm) was measured to be around 10% for a polished surface and 20–30% for a rough surface of powder layer at normal incidence. Therefore, the reflection coefficient of 316L stainless steel at 1.07 µm in the case of LPBF process can be estimated to be around 0.25 (we appended this parameter in the last row of the Table 3). However, this is only an estimate suitable for our particular case, and the actual reflection coefficient may vary depending on the specific conditions of the laser-stainless steel interaction. In most cases, the absorbance is linearly dependent on temperature. However, we are considering the total amount of thermal energy applied to the powder layer when heating it up from room/chamber temperature to its melting point, so the average “effective” absorbance will be approximately 1 − R = 0.75.

The lattice geometry to print to shown in the right half part of Fig. 1 doesn’t need for additional geometry of supports, as its seven “legs” are sufficient for powder layers not to fall down. The absence of supports makes the preprocessing stage simpler, and we do not need to mesh the supports. Instead, we just define Dirichlet-type boundary conditions on the “foots” of a printed part where they connect to the base (or substrate) at constant room temperature of 300 K. Other mesh boundaries are perfectly insulated, but it is also possible to define their convection coefficient. This is about the thermal conditions. And for mechanical boundary conditions we are obviously setting fixed zero displacement on the “part-to-base” faces and leave other faces free deformable. However, to simulate the cut-off stage when the fully printed part is detaching from the base platform, we change boundary conditions of previously fixed points of deforming mesh in such way, so just a little amount of them remain fixed (we call this “partially fixed”), and others are becoming free.

Now let’s provide all real-life laser processing settings converted to solver settings according to expressions (1–3) and laser processing mode specified in the Table 1. Laser exposure time on each powder layer, as well as the temperature of the deposited layer at the laser beam spot, have been calculated from the laser trajectory and speed. Powder deposition time, its cooldown time (t_dwell_real_) and fusion time (t_preheat_) have been estimated the same way according to geometric size of the printed part and thickness of each real layer of powder grains. Then these important values have been upscaled to supply temporal settings for super-layers of deformable mesh deposited one by one. Recall, that the super-layer may consist of some real powder layers to speed-up calculations. The super-layer-related times are the key input parameters of the solver and are listed in Table 4.

**Table 4:** Real-life and corresponding input parameters of the thermal-structural solver when virtually printing the “lattice” (or “comb”) geometry.

Parameter	Value	Meaning
T_preheat_	1645 [K]	A temperature that laser produces on the powder layer to melt/sinter it. In fact, this is a deposition temperature of any super-layer of the mesh
N_single_in_a_super_	55	How many real powder layers the super-layer of the mesh consists
N_super_	10	Count of horizonal super-layers of the mesh
dt_sol_	100 [s]	Solution step time (both in C# and Ansys)
t_preheat_real_	13 [s]	Average laser exposure time on each real deposited layer of powder
t_dwell_real_	22 [s]	Average cooling time of each real deposited layer of powder before to deposit the next layer
t_preheat_	715 [s]	Laser exposure time on each super-layer of the mesh
t_dwell_	1210 [s]	Cooling time of each super-layer of the mesh before to deposit the next one
t_cut-off_	20,213 [s]	A time to partially detach the printed part (the built and cooling stages should be completed) from the base changing the boundary conditions of lower mesh node points from “all fixed” to “partially fixed”

Our solver calculates all these parameters and automatically activates new super-layers of the deforming mesh according to time spans of preheat and dwell stages. It also automatically changes the boundary conditions when it is time to cut the completely printed and cooled-down part off the base. However, to achieve similar functionality in a basic tandem of Ansys’ thermal and structural FEM solvers, we need explicitly pick each layer of the mesh and pre-define its thermal and mechanical boundary condition with respect to time, so they can “birth” and heat-up every (t_dwell_ + t_preheat_) span of simulation time. For a relatively low number of mesh layers in the lattice geometry, we can select each layer’s nodes visually by hand, and it’s relatively straightforward. For more complex geometries, this routine become not so easy, and we need to select nodes via Ansys “worksheet” functionality. This step will be described further for the turbine geometry.

**Figure 10:**
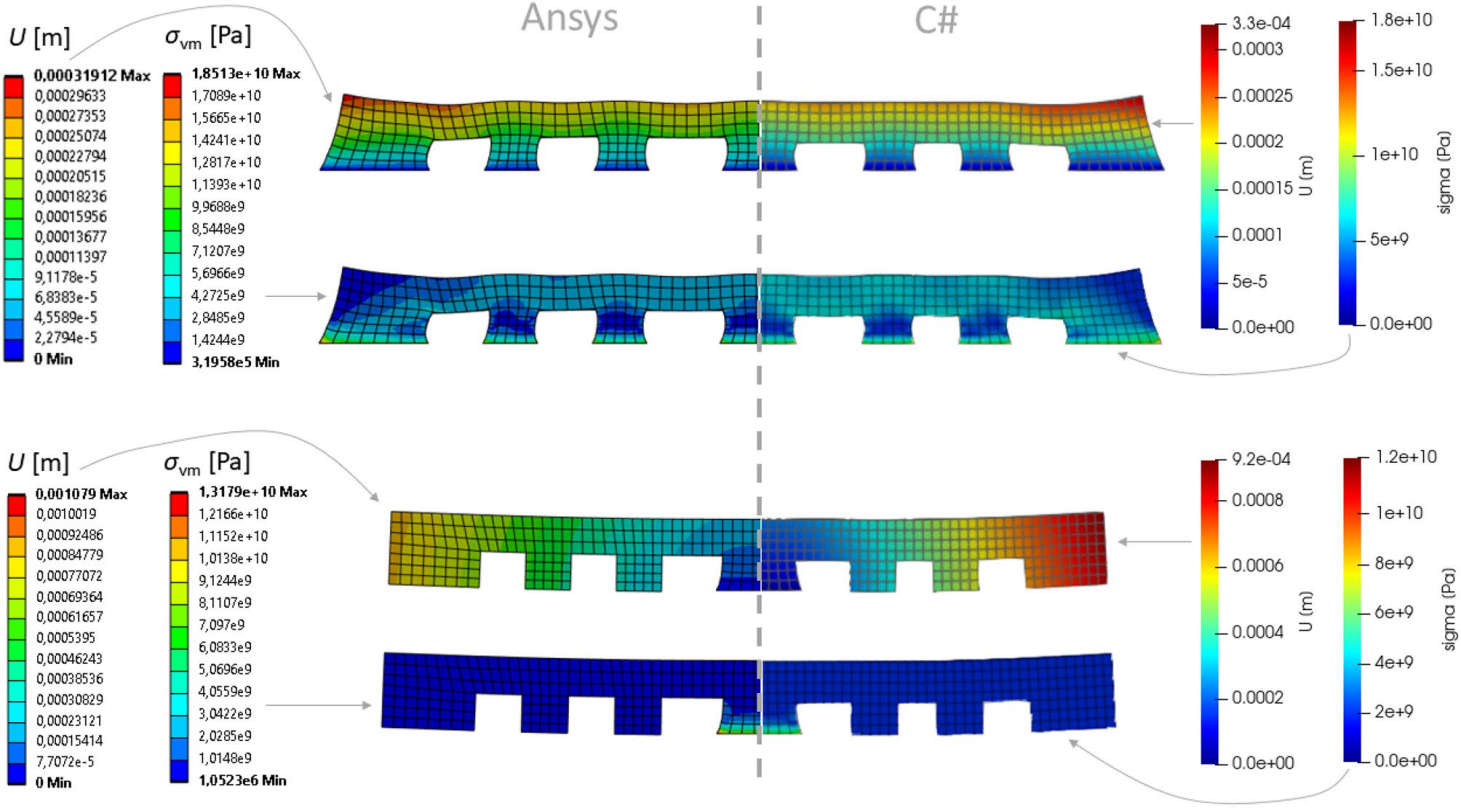
Solution results (displacement magnitude) of 3D printing simulation of the “lattice”. Left: Ansys thermal + structural; Right: custom C# solver (visualized in ParaView); Top: before cutting off from the base; Bottom: detached from the base.

Every time step, the solver converts deforming mesh into hexahedral blocks and write nodal values and grid structure to VTK file format^27^ to be opened then in a free ParaView scientific data visualizer. In the ParaView we apply the “warp by vector” geometry modifier to move each node of a grid according to computed displacements in these nodes. This allows us to visually compare results produced by Ansys Workbench visualizer and by ParaView side-by-side (Fig. 10). For the sake of clarity, all deformations have been upscaled, because the absolute values of displacements are relatively small (refer to the color axes represented in meters) and do not exceed 0.33 mm for both solvers. Thus, the nodal displacements of the mesh were upscaled by a factor of 10 to enhance the visibility of distortions.

There is a noticeable good qualitative and quantitative agreement (Fig. 10 and Table 5) of the spatial distributions of displacements in the entire computational domain with minor deviations, although the methods of solution are fundamentally different: FEM-based Ansys and Largargian-particle-based custom solver in C#. The comparison was also made for a purely elastic material, for which, unlike Table 3, the value of the Tangent Young’s modulus when exceeding the local stresses of the Yield strength remained the same as the base Young’s modulus from the fourth row of the table. In both cases, the relative deviations of the maximum displacements for Ansys and C# do not exceed 4% (the last column of a Table 5), while the accuracy in the elastoplastic is 0.7% lower (3.2% vs 3.9%), which, apparently, is explained by the rough explicit Euler time integration scheme, as it is shown in formula (25).

**Table 5:** Relative discrepancy between maximal displacements after the cut-off, obtained for the “lattice” geometry in Ansys and custom solver.

Model	Ansys [m]	Custom C# [m]	Absolute δ [m]	Relative δ
Elastic	3.19*10^–4^	3.3*10^–4^	0.000010	0.032
Elastoplastic	3.27*10^–4^	3.4*10^–4^	0.000013	0.039

All simulations have been performed using single thread on reference system equipped with Core i7-3770 CPU and 16 Gb of RAM, Windows 10 64-bit. Surprisingly, the simulation time on custom solver appears to be lesser than the Ansys’ time (Table 6): Lagrangian solver is about 1.5 times faster. Probably this circumstance is caused by the way Ansys and custom solver solve the governing equations. As a FEM-based solver, Ansys assembles a global FE matrix consisting of [N x N] elements, where N is the mesh nodes count. Full mesh-wide global matrix assembly from a set of local cell-related matrices should take significant calculation effort, and the solution of the obtained linear system of algebraic equations is a hard task as well. In our Lagrangian solver, in contrary, there is no need to build any matrices at all. Instead, we iteratively propagate thermal flow and stress-induced displacement from one link to all others distributed over the deforming mesh. This approach may look like the iterative Gauss–Seidel relaxation algorithm to solve systems of linear equations, although here the system is defined implicitly, in an algorithmic rather than mathematical way (expressions 16–26).

**Table 6:** Relative performance measured as the time for simulations to complete.

Task	Ansys [s]	Custom C# [s]	Relative speed-up > 1: C# is faster < 1: C# is slower
Mesh generation	12	57	0.2
Elastic simulation	1136	608	1.8
Elastoplastic simulation	1331	961	1.38

The bottleneck of our Largrangian solver is its mesh preprocessor. Voxelization stage of the interior enclosed with a given faceted STL surface and mesh generation time is about 5 times (0.2 in the last column of Table 6) slower than the Ansys does. This is probably caused by additions operations to build not only deforming mesh cells, but also add links inside them along their edges, crossing on their faces and following inner diagonals, as it is schematically depicted in Fig. 4d.

**Figure 11:**
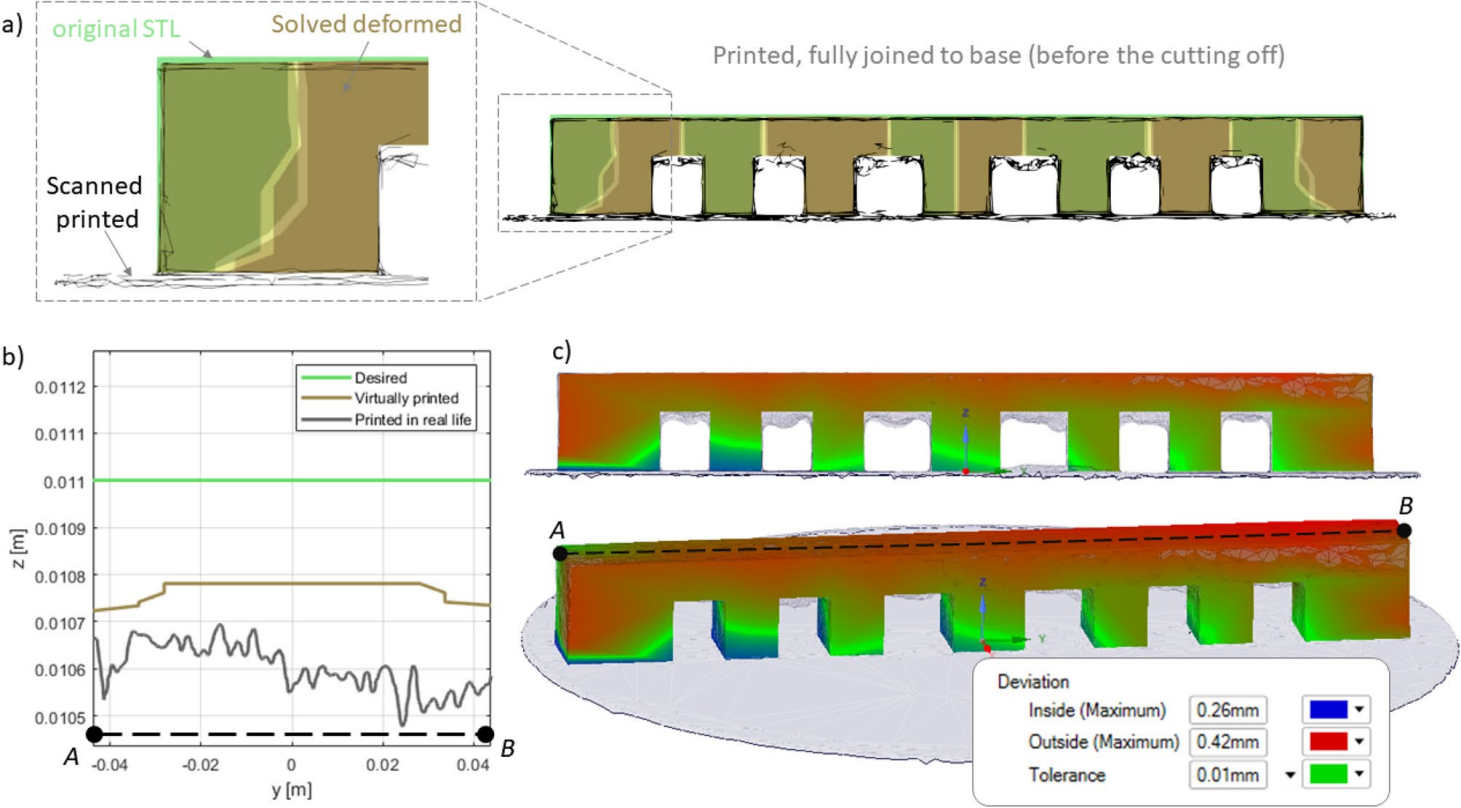
Printed “lattice” geometry connected to base. Comparison of solved and real-life printed (and scanned) surface meshes. (**a**) Comparison in ParaView: original geometry (green), deformed geometry produced by custom C# solver (red blended with green creates a golden-brown color), and scanned (black outline); (**b**) Deviation of the predicted 3D surface geometry from the actual printed one; (**c**) Surface profiles width-averaged along the longitudinal direction (A-B line) sampled from the top side of the printed part.

Once the calculations have done on deforming mesh, its final deformation is projected onto initial STL surface mesh as described in the Sect. “Shape compensation algorithm” of the “Materials and methods” chapter. This allows us to compare predicted deformations to ones obtained in a real-life 3D printing experiment under equivalent operating conditions. Figure 11 demonstrates semi-transparent green original STL mesh, semi-transparent virtually printed-out deformed mesh still before the cut-off stage (computed by means of our custom solver) and a black outline wireframe of scanned mesh after its real-life printing (as it is shown in a top part of Fig. 2). Although being colored in red, the deformed mesh looks mostly “olive-brown” because of the way the ParaView blends semi-transparent colors. Thus, overlapped red and green zones will look olive. Visually, it is clear (especially in a zoomed-in frame of the Fig. 11a) that the predicted shape deformation (olive-red) is close to the printed one (black wireframe). To estimate numerical values of discrepancies, we compare these meshes in the SpaceClaim that is basically Ansys’ built-in CAD and geometry processing system. The both faceted meshes (solved and scanned-out after its printing) are per-node compared and their mutual deviation from each other is pseud-colored from blue to red corresponding to inner and outer deviations (Fig. 11c). The scanned one has its own coordinate transform that commonly differs from the coordinate system of original and virtually printed surface meshes. That is why it is not easy to perfectly snap these two meshes. We use the semi-automatic “Align” tool of the SpaceClaim to force the scanned and predicted meshes to be located at similar points in 3D space. Visually these meshes match relatively good, and their deviation is in a [0.26–0.42] [mm] range, which correspond to about 2% of relative deviation keeping in mind, that the model height is 11 [mm]. This discrepancy is enough to claim that the solver is capable to predict shape distortions and residual deformations of a model after its LPBF based 3D printing.

**Figure 12:**
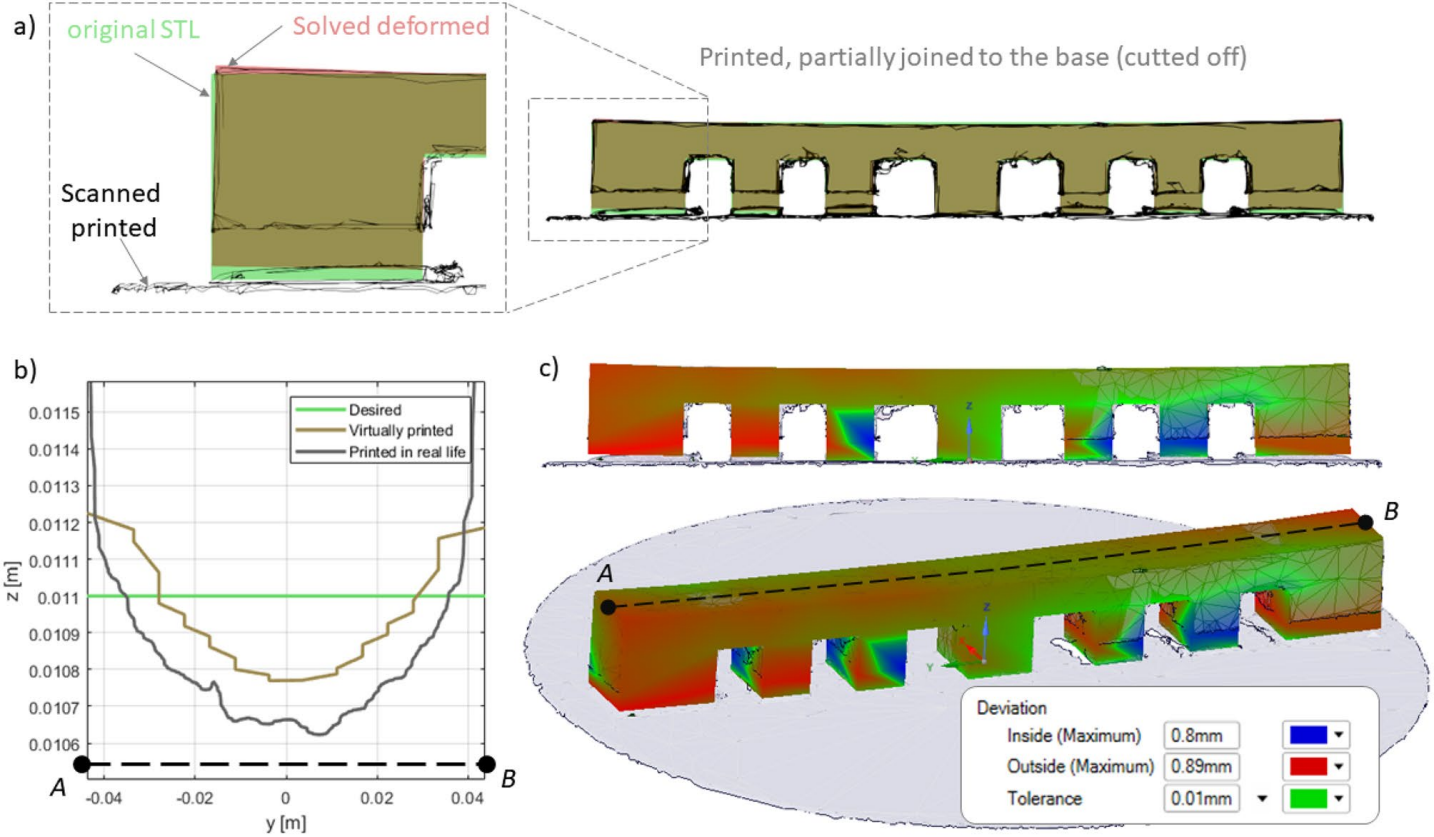
Printed “lattice” geometry after it cutting off the base. Comparison of solved and real-life printed (and scanned) surface meshes. (**a**) Comparison in ParaView: original geometry (green), deformed geometry produced by custom C# solver (red, blended with green), and scanned (black outline); (**b**) Deviation of the predicted surface geometry from the actually printed one; (**c**) Surface profiles width-averaged along the longitudinal direction (A-B line) sampled from the top side of the printed part.

For the purpose of visually comparing virtually and actually printed real-life surfaces, we process the STL files in MATLAB by extracting all coordinates of their vertices. We then identify the vertices with the highest vertical coordinates, representing the top side of the printed part. These selected coordinates are then sorted along the A-B line (refer to Figs. 11 and 12b,c) while being averaged over the part’s thickness (in a direction of the side normal to the figure or screen). Averaging is necessary when sampling the scanned real-life printed part because its shape contains significant noise. To mitigate this, we apply a low-pass filter to the vertical coordinates of the STL vertices’ top side. This filter attenuates high-frequency components while allowing low-frequency components to pass through, with the cut-off frequency set to 0.025. Additionally, we mathematically average the vertical components of the vertices of the virtually-printed part. This conversion from 3D planar coordinates at the top of the part to 2D coordinates along the A-B line simplifies plotting all these vertices in a single 2D figure, as depicted in Fig. 11b.

Low-pass filtering typically causes a decrease in the signal, resulting in the filtered-out dark-gray curve (representing the profile of the top side of a real-life printed part along the A-B path) appearing slightly lower (see the vertical axis of Fig. 11b) than the profile of the virtually printed (simulated) part depicted in golden-brown color. In this case, the part is still connected to the base, and all its legs are on the ground, causing its top side to appear slightly lower than the desired profile (depicted in light green curve) due to residual deformation and shrinking of the surface as it cools down to room temperature.

Now let’s see how the printed part distorts after detaching of the most of its “legs” from the base. When considering a part that has been cut off from the base, the residual stress tends to relax, causing the part’s longitudinal sides to bend and raise up. This de-stressing effect leads to the profile of the top side of the part being higher at the opposite sides than the original desired profile. This is clearly observable in Fig. 12b for both the simulated (golden-brown) and real-life (dark gray) profiles of the printed part.

Similarly to Fig. 11, we made a comparison of the surface mesh predicted by our solver (Fig. 12a, red) and the scanned one after its printing (Fig. 12a, black wireframe). Again, as expected, they both diffs from the original STL surface (Fig. 12a, green), but their difference from each other is not visually too large. However, when we are comparing them in the SpaceClaim, the deviations (Fig. 12c) are about 2.6 times larger (~ 0.8 [mm]) than in case when the model was not yet detached from the base (~ 0.3 [mm]). This is because it was a tricky task to detach the printed model from the base in real life conditions: and one can see, that six legs of the model have been cut slightly higher rather than directly at the level where they connect to the base. That is why all real-life detached legs of the scanned mesh is shorter than the ones of predicted mesh, and that’s why the deviation is so high. In fact, if not to consider the bottom parts of these legs, the deviation should be about the same as it was before the cutting-off stage. Both the printed and predicted surfaces demonstrate symmetric lifting of their left and right corners when detached from the base.

So, for now, the solver has successfully proved its capabilities to predict shape deformations of 3D printed parts and their buckling when detaching from the base. The next step of verification is to perform the same simulation on more complex geometry of a turbine.

## Results and discussion

Here, we deal with 3D printing simulation and corresponding real-life experiments of parts with significantly more complex shapes than the test parts (1D rod and a comb, or lattice) described previously in Subsection 4 of the “Materials and methods” chapter. The material properties are the same as they were for the lattice part. In order to test the solver in real-life conditions, we consider relatively complex shapes: a turbine (printed without supports) and an impeler printed with full supports geometry, as this shape contains hanging blades. The goal is to properly predict the shape and size of the printed part and then to correct its geometry in order to minimize distortions.

### Deformation of a complex shaped part printed without supports before and after the base removal

In this chapter we are dealing with the simulation (Ansys and custom solver) and real-life experiments of 3D printing of a turbine geometry (Fig. 1, left) of the same powder material (316L stainless steel). The laser parameters remain the same (Table 1) as for the “lattice” geometry, and the model-specific build parameters are dependent on its size and thus differ from the ones (Table 4) used when printing the lattice. Table 7 contains printing conditions and solver settings for the turbine mesh, and the meaning of each parameter is described previously in Table 4.

**Table 7:** Real-life and corresponding input parameters of the thermal-structural solver when virtually printing the “turbine”.

Parameter	Value	Parameter	Value
T_preheat_	1798 [K]	dt_sol_	100 [s]
N_single_in_a_super_	38	N_super_	18
t_preheat_real_	16 [s]	t_preheat_	617 [s]
t_dwell_real_	43 [s]	t_dwell_	1677 [s]
t_cut-off_	43,344 [s]		

**Figure 13:**
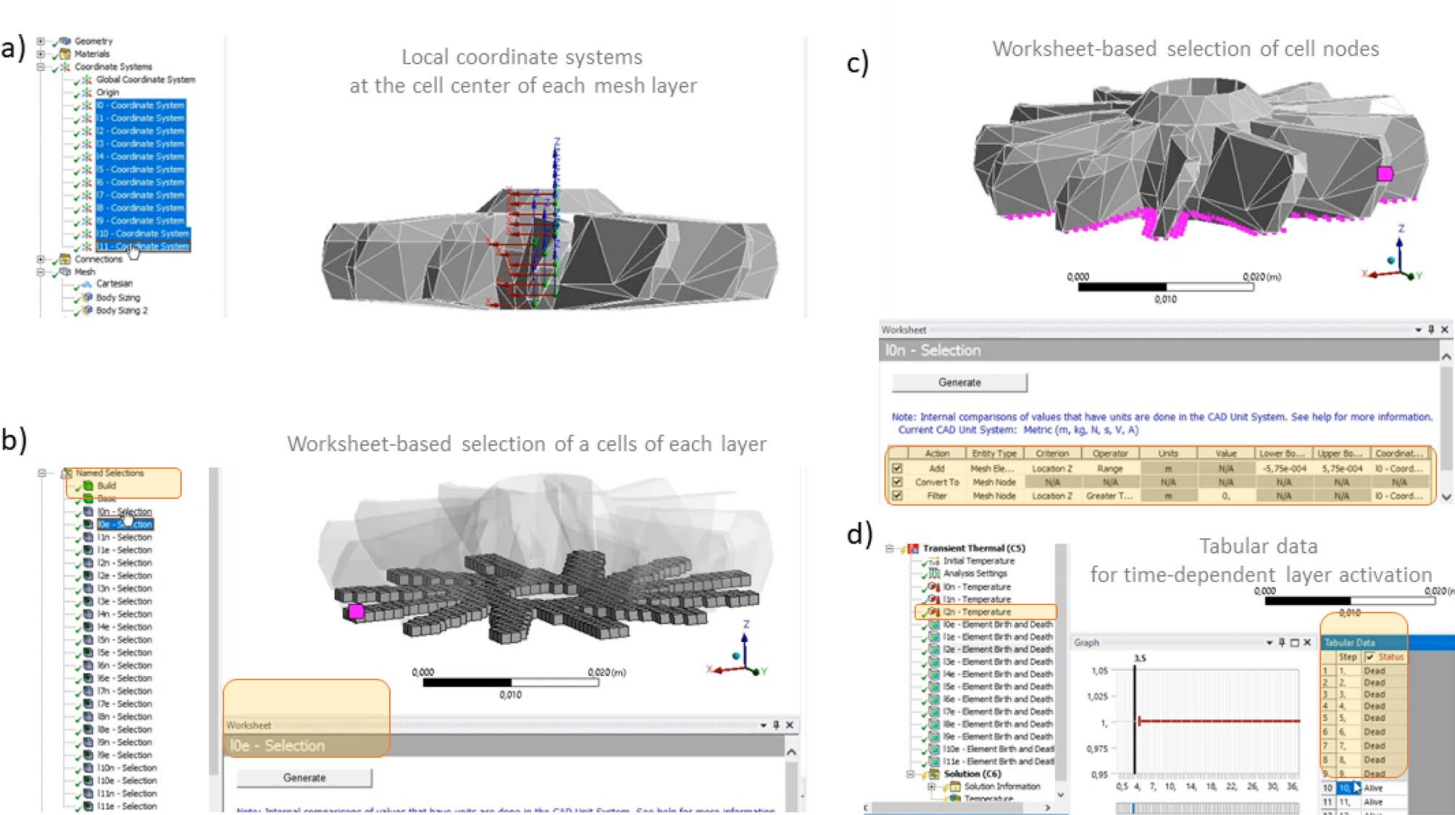
Manual settings for per-layer activation and thermal boundary conditions to emulate 3D printing in standard Ansys Thermal-Structural analysis. (**a**) Creating local coordinate systems; (**b**) Cell selection for each layer; 3) Nodes selection; 4) Element birth and death activation.

Let us describe how we should select a mesh in the Ansys layer-by-layer. For the lattice, the node section and setting their BCs was straightforward. For the turbine mesh, this process is more complex and should be semi-automated based on named selection and “worksheet” approach (depicted in Fig. 13) in the Ansys Workbench. Ansys does not automatically set-up boundary conditions of each mesh layer depending on their instants of deposition (according to t_preheat_ and t_dwell_), so we need to define them explicitly in manual way. Once the Cartesian-like FEM mesh has built, we create a series of local coordinate systems at the center of each layer (Fig. 13a). Then, for each mesh layer (let’s say, layer 5) we create a series of “named selections” of hexahedral cell elements of the mesh (“l5e”, where “e” stands for “element”) and element nodes (“l5n”, where “n” stands for “node”) that are located at element’s top face (Fig. 13b,c). Element selection is based on Ansys’ “worksheet” approach, when a selection is composed of a set of rows with combo-box GUI selectors acting as a series of mesh-related filters. In fact, once the local coordinate system of each layer has defined, we select all cells which vertical z coordinate is in a cell’s height distance relatively to z coordinate of the local system. Thus, way we can select nodes of particular mesh layer.

Then we convert mesh elements to nodes and select the greatest ones as it corresponds to element’s top side. All these three steps are marked in orange box in Fig. 13c. Finally, we activate/deactivate mesh elements and set/unset T_preheat_ temperature at their nodes with respect to layer fusing time and its deposition time, obtaining a table of “steps” as depicted in Fig. 13d.

**Figure 14:**
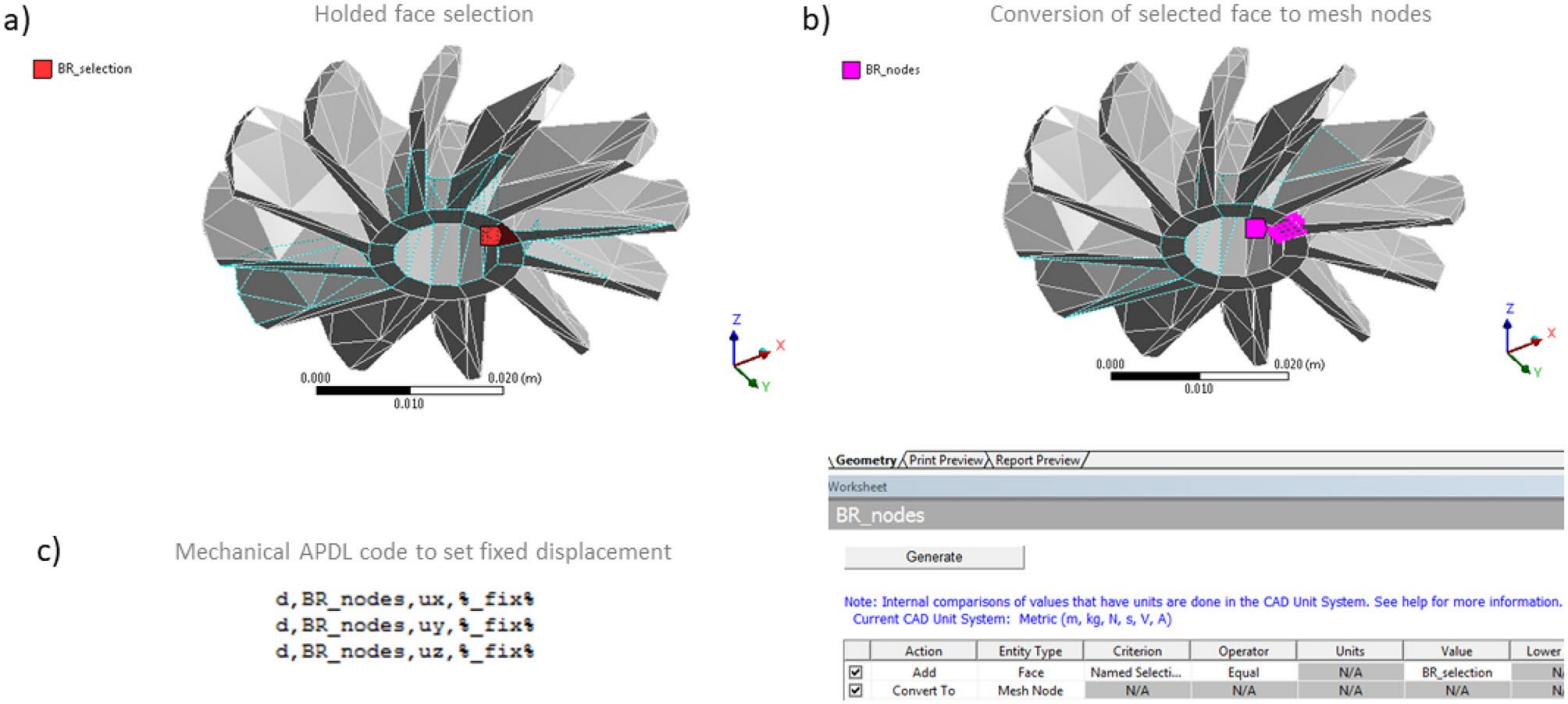
How to override fixed displacement boundary conditions for a region that remain held (connected to the base) after the cutting-off stage.

To detach the model from the base at the final step we append the Ansys Mechanical APDL code as it is shown in Fig. 14a, where “d” stands for “nodal displacement”, and “ux”, “uy”, “uz” are the components of displacement vector. The mesh nodes that must remain connected to the base permanently have been marked (independently of the mesh resolution) based on a manually selected face of the surface geometry (Fig. 14a). Selected face is converted to related mesh nodes (Fig. 14b) uses the “worksheet”-type of the named selection. At the base removal (BR) solution stage, all mesh nodes have been changed to “free” boundary conditions, except for the previously selected mesh nodes called “BR_nodes”. The displacements of these nodes correspond to a “fixed” boundary condition that can be applied through the Mechanical APDL code snippet shown in Fig. 14c.

**Figure 15:**
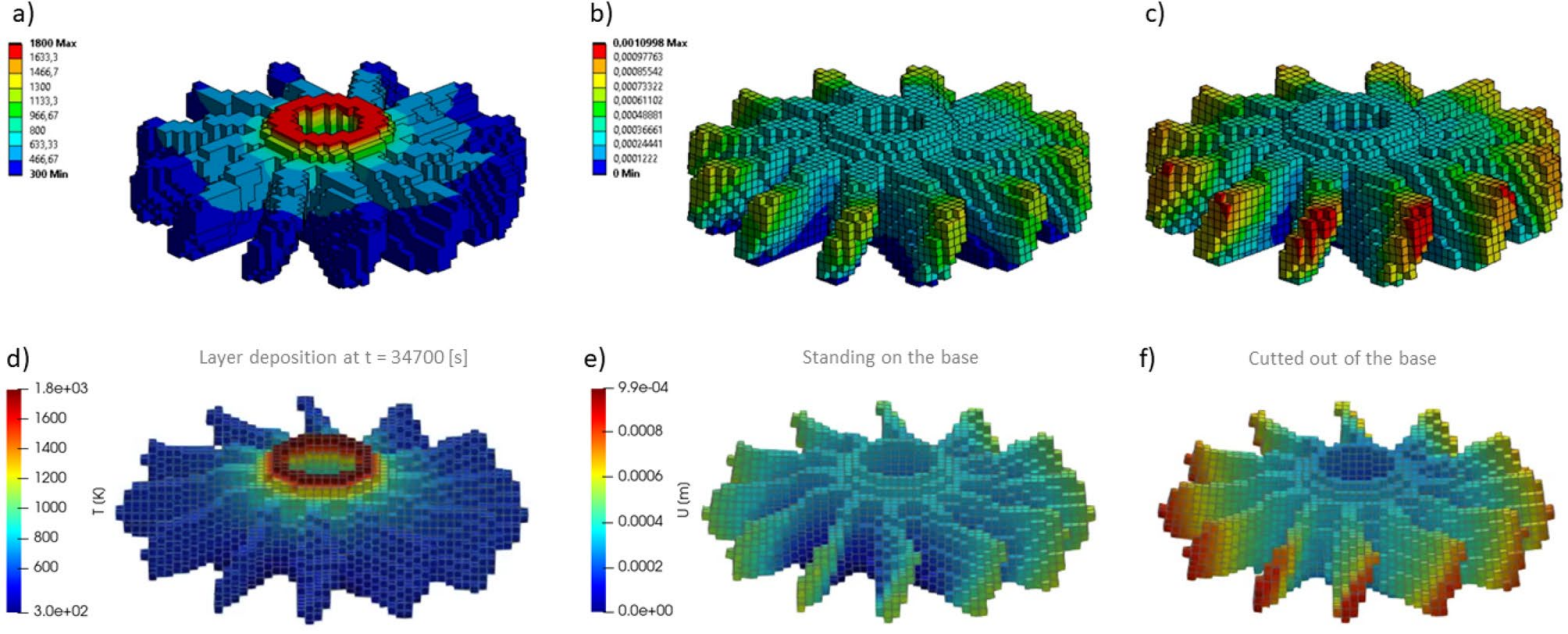
Virtually printed turbine mesh: temperature and displacements calculated in Ansys (**a**, **b**, **c**) and in custom Lagrangian solver written in C# (**d**, **e**, **f**). For the sake of convenient visual perception all displacements have been upscaled (× 5), however color axes remain unchanged.

As the turbine is more complex than its predecessor lattice, the thermal-structural dynamics within its volume may be less stable. In particular, the solution in the Ansys has converged only when considering the structural dynamics in the quasi-stationary approximation and introducing a damping of 25% for the stiffness and mass coefficients. The results of simulations under equivalent initial and boundary conditions, mesh, and solver settings are presented in Fig. 15.

Here we provide temperature distribution (Ansys: Fig. 15a, C#: Fig. 15d) in the last deposited layer of the mesh at the very end of its preheating by the laser source. These two images show how the temperature propagates deep inside the mesh volume. Nodes at the mesh-to-base interface have a fixed ambient temperature.

The rest part of the Fig. 15 shows nodal displacement magnitude (Ansys: Fig. 15b,c, C#: Fig. 15e,f) before and after the cutting from the base. Note that here (as in the case of lattice geometry) it was not necessary to mesh the geometry of the base itself (because there are not any supports), instead we just hold these nodes at fixed temperature and displacements until they are connected to the base. When the mesh is totally cooled down to the ambient temperature (43,344 [s] according to Table 7), almost all bottom nodes become free deformable, and residual stresses at these nodes raise the value of final displacement on the tips of turbine’s blades (compare b–c and e–f). Maximal displacements obtained in the Ansys (1 [mm]) and in our solver (0.9 [mm]) mostly corresponds to each other.

**Figure 16:**
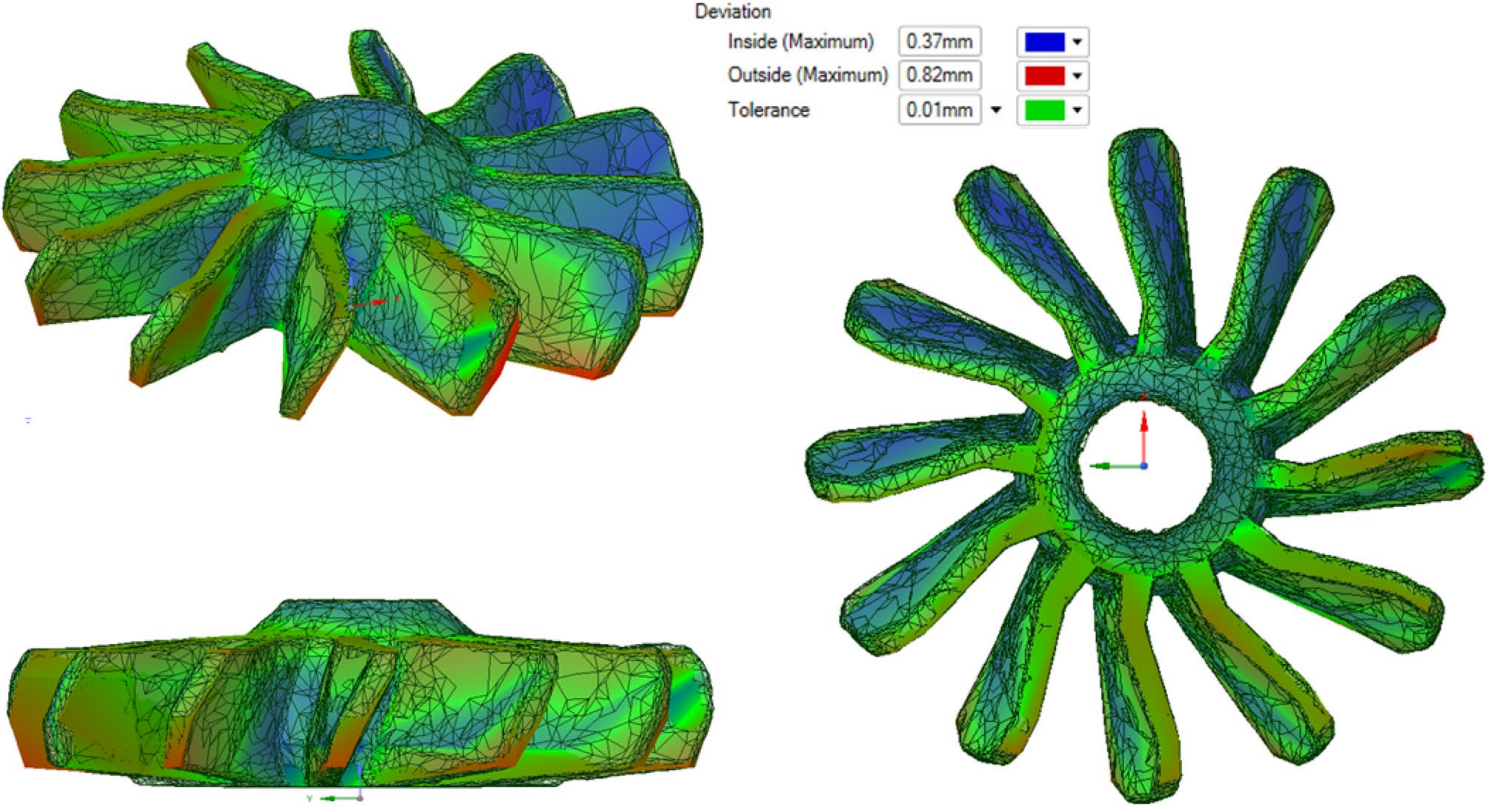
SpaceClaim-based comparison of scanned and virtually printed meshes of the same turbine after it is cutting out of the base platform.

The next step is to compare the scanned mesh of real-life printed turbine to the one obtained via the projection of initial surface STL mesh onto the final state of deforming mesh at the end of computations. Per-node discrepancies (Fig. 16) of scanned and simulated meshes, as previously, are calculated in the Ansys SpaceClaim using the “Measure” tool.

Here we see that the level of coincidence between real-life and simulation is higher than it is in the case of printing the lattice mesh (2%). Average deviation is about 0.6 [mm], which causes a relative deviation of 4% (turbine height is 14 [mm]). The reason turbine has 2 times worse coincidence to experiments may be in that it is harder to snap/align the scanned turbine mesh to the simulated one (as their coordinate systems do not match, because the scanner’s coordinate system is arbitrary). However even 96–98% of coincidence should be enough to use the solver in cases where residual displacements of the printed part exceed the error range of 2–4%.

### Residual deformations after printing (with supports) of compensated geometry derived by means of simulation

The last but not least test is to print even more complex “impeller” geometry meshing its supports and the base, and then auto-correct of shape deformation (ACSD), print the corrected shape again and check whether it helped to reduce deviation of printed part from the original (desired) one. For this task, the simulation has been performed just on custom Lagrangian solver, because it has been verified in two previously discussed cases. The second reason Ansys was not used is because it is difficult to perform manual selection of layers and setting of their thermal and structural conditions for every time step of the simulation, especially for two meshes: the original and the CSD-compensated one.

**Figure 17:**
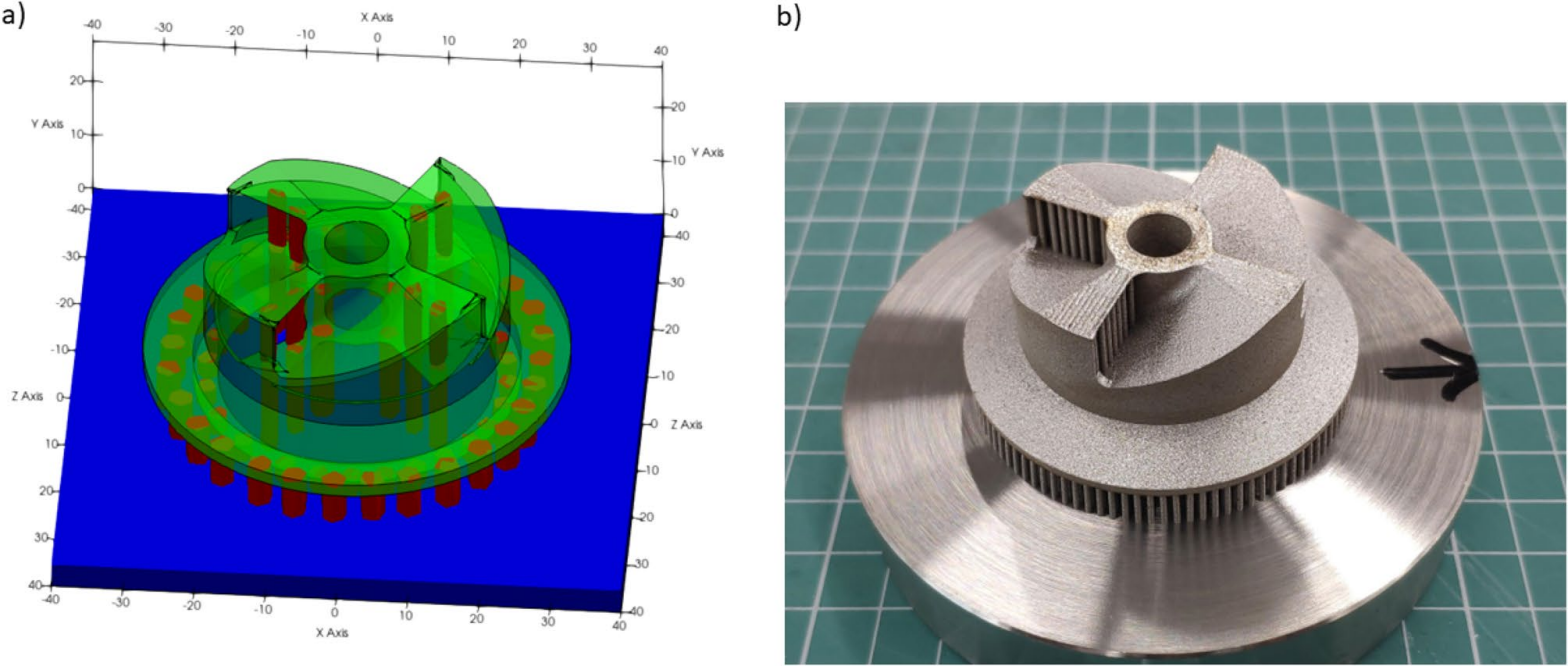
A geometry of the impeller (axes dimensions are provided in millimeters). (**a**) Surface mesh (green) with the base (blue) and simplified supports geometry (red); (**b**) Real-life printed part with detailed geometry of the supports.

An “impeller” geometry has also a larger size (Fig. 17a) than its two predecessors. This is needed, primarily, to make shape distortions essential to compensate them further. For small printed parts, obviously, the shape distortions will be equal to about the scanning error. Large parts, in contrary, may suffer from notable distortions—and here is the field when our solver can help. Here in the Fig. 17a we draw the desired geometry is semi-transparent green color to prove that this case needs to model for supports as well, as it has hanging body parts especially in a zone of inner propeller blades. Because the key role of supports is a fixing of the powder layer and an effective heat sink, to diffuse-out a heat from the layer to the base, we may define their geometry in simplified way (compare the supports in Fig. 17a, b) in order to decrease the computation effort. However, they still can perform their primary functions.

The powder material and laser parameters are the same as depicted in Table 1 and in Table 3 and these values have been converted according to the mesh layers count (20) to the deposition duration of each super-layer and its cooling (dwell) time. At the cut-off time of 60,000 [s], all support-related cells (and links) of a deforming Lagrangian mesh are deactivated, except for a small part marked as permanently fixed, as was done in two previous test cases. The test case for the “impeller” geometry consists of the following steps (these key distortion correction steps are also described previously and are schematically shown in the Fig. 7):

1. Virtual 3D printing of the original STL geometry in our Lagrangian thermal-structural simulation software, followed by comparison of its shape after detaching from the base with the reference original shape;
2. Real-life 3D printing of the original (reference) STL geometry, followed by 3D scanning of the part to compare its shape with the original (desired) shape;
3. The ACSD stage to predict a “corrected” STL geometry that, once printed, will perfectly match the desired original one;
4. Virtual 3D printing of the corrected (deformation-compensating) STL geometry, followed by comparison of its shape with the reference design;
5. Real-life 3D printing of the test part according to the compensated STL geometry, followed by its 3D scanning to compare its shape with the reference.

**Figure 18:**
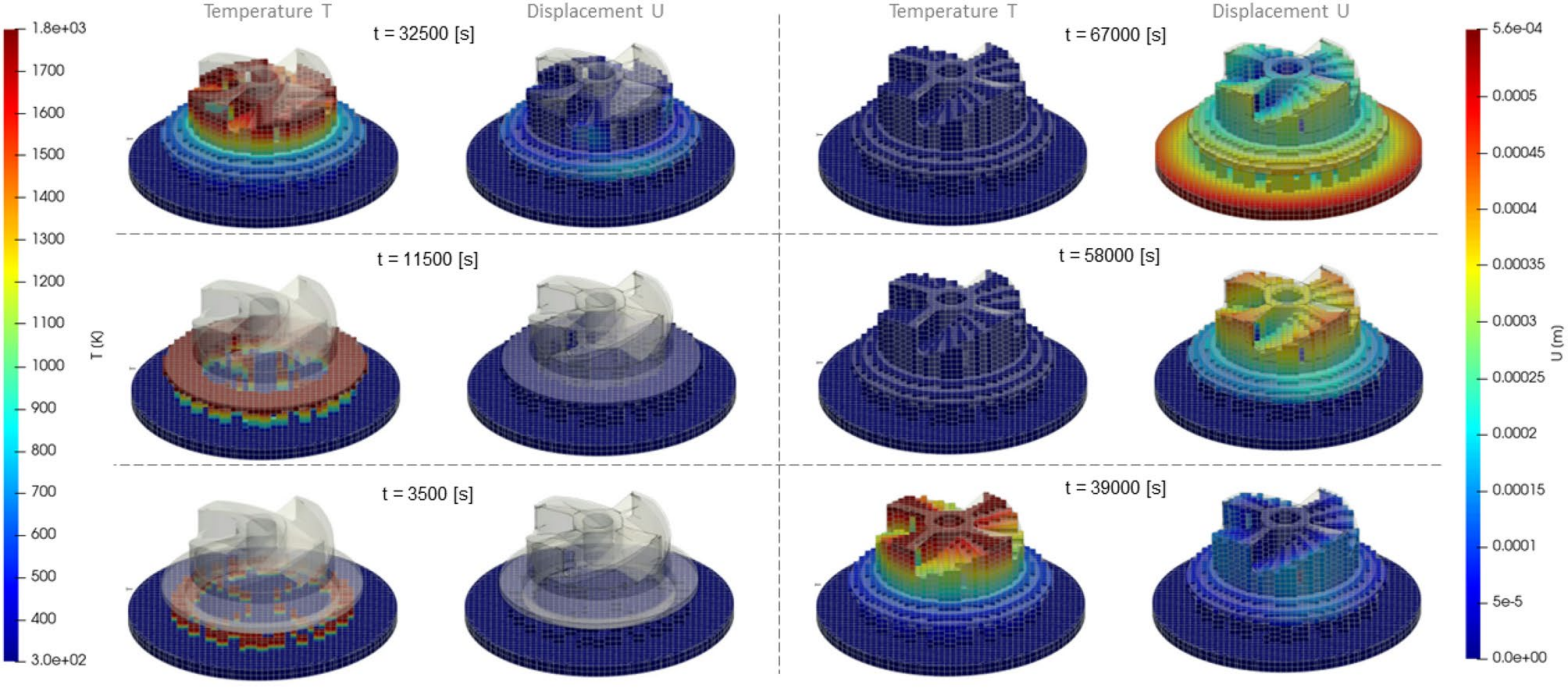
The results of simulating 3D printing in a custom Lagrangian solver at various time steps. The displacement scale is × 1.

Let us consider the first step. Here, we are modeling a process of 3D printing of the original STL mesh as it should be. Figure 18 illustrates this process at different time steps. The original STL mesh is presented in each frame as a semi-transparent overlay to visually check what is occurring and how the deforming mesh matches the desired shape.

At a time of 39,000 [s] since the beginning of 3D printing, the last super-layer of the mesh has been deposited. The mesh is then left to cool down, while still connected to its supports and the base. At t = 58,000 [s], the cooled mesh has reached ambient temperature, and the maximum displacement magnitude is not greater than 0.4 [mm]. After this, the mesh nodes corresponding to the supports and the base change their boundary conditions from fixed Dirichlet to thermal insulation Neumann-type conditions and the free-surface condition. This results in an increase in the displacements of the base and the supports compared to the frame at the earlier time step, which no longer affects the part’s body itself.

As can be seen, the deformed mesh at its final stage (base and supports removed) at 67,000 [s] has a shape proportional to the original STL but differs in size. The printed model appears to be smaller than the desired original one. To address this, the process goes ahead to the second ACSD stage and predicts the corrected STL surface that will compensate for size discrepancies even if the printing conditions remain unchanged. Intuitively, if the initially printed (at the first stage) shape is smaller than the original one, the corrected geometry should be slightly larger than the original surface mesh. Once printed again, this enlarged corrected model will then shrink, so its size after the printing process has finished will be remarkably close to the original reference mesh. The shape correction is performed according to Eq. (30).

**Figure 19:**
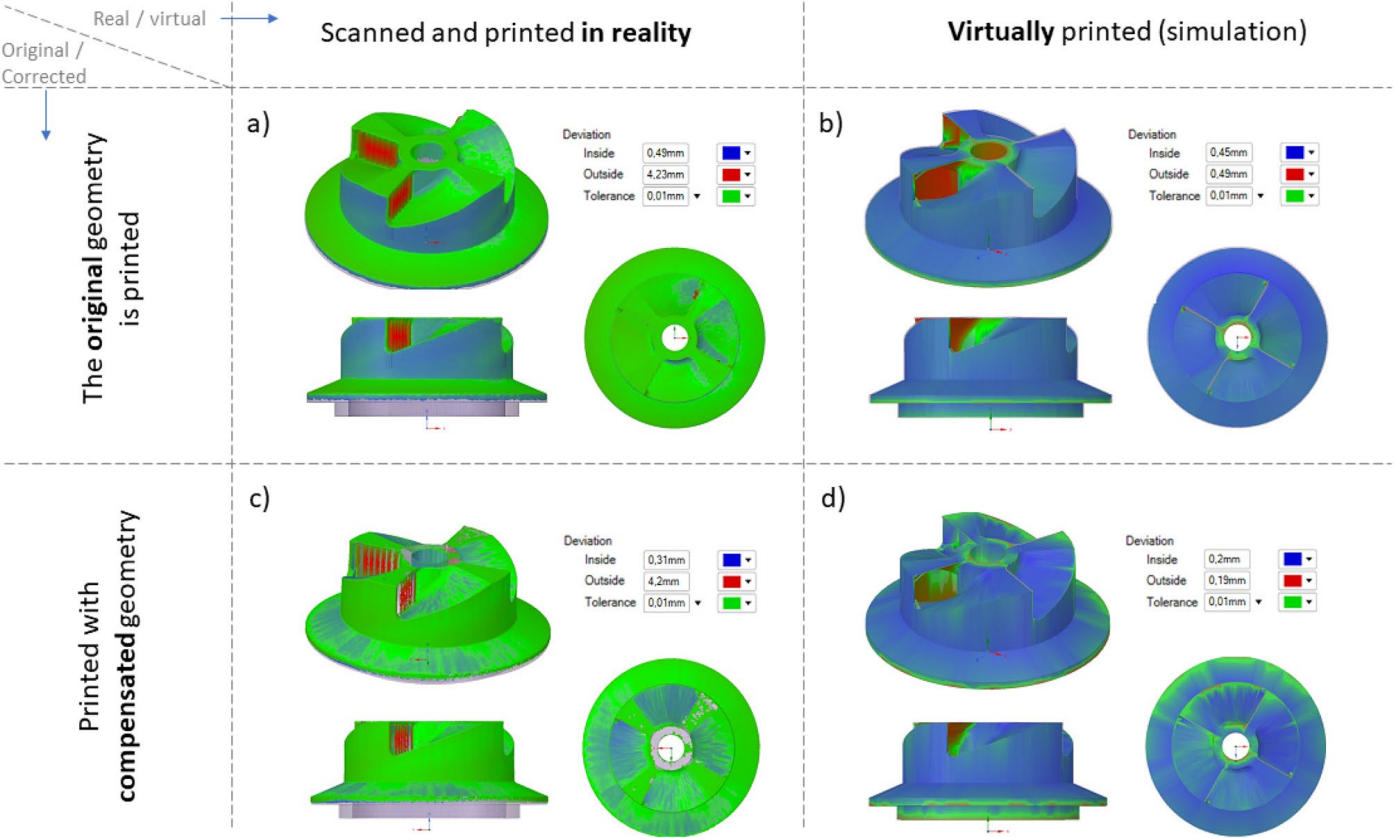
Deviations of the printed parts from the original geometry that is shown as a violet overlay in each frame. (**a**) Real-life printed original geometry; (**b**) Virtually printed original geometry; (**c**) Real-life printed corrected geometry; (**d**) Virtually printed corrected geometry.

For clarity, we have collected the results of the remaining three steps into a single “table”-like diagram in Fig. 19. The top row of frames corresponds to the real and virtual printing of the original reference geometry of the impeller. The two frames at the bottom row correspond to the real-life and virtually printed corrected geometry. Note that the geometry correction step runs immediately after the completion of the first virtual printing of the original geometry. Each frame of this “table” consists of a point-by-point (vertex-by-vertex) comparison of the original faceted mesh, which is colored uniformly in violet, and the printed one. The comparison, as before, was done in the Ansys SpaceClaim software. For the real-life printed parts, we load their 3D scans semi-manually adjusted to match the coordinate system of the reference mesh: in Fig. 3 you can see that the axis triad is located at an arbitrary point and is slightly rotated, indicating the coordinate system of the scanner. For the virtually printed part, we load the surface mesh projected onto the distorted deforming Lagrangian mesh. Note that the exported deformed virtually printed part does not include the support geometry in the file, whereas the scanned one has supports. These supports make shape deviation measurement inaccurate when comparing the scanned real-life printed geometry to the original one, which does not have supports. Therefore, only the “inside” deviation is low, as it does not consider the supports, in contrast to the “outside” deviation. For example, in (a), the “inside” deviation is 0.49 [mm], while the “outside” deviation is 10 times larger (4.23 [mm]), as it considers the scanned geometry of the supports, which are colored red.

The table allows us to compare simulated and real-life results and how they correspond to each other. The comparison is indirect, as to compare, for example, the really and virtually printed original part (Fig. 19a, b), we need to subtract their deviations from the original mesh. However, note that the only data we should consider is the “inside” deviation, as described above. Specifically, let us compare how (a) and (b) correspond to each other. To do this, we compute the absolute difference between the “inside” deviation from (a) and the “inside” deviation from (b): (0.49–0.45) [mm] = 0.04 [mm]. This is how the displacements of the virtually printed mesh differ from the one printed in real life. The printed part is about 40 mm in width, so the relative error between the numerically simulated and experimental results is about 0.1%. This error is much less than the errors obtained on smaller parts, as the larger the printed part is, the lower the 3D-scanning error.

The second valuable property of this table is a direct dataset, showing how each printed part differs from the original reference design. Once the original geometry is printed (in real life or virtually) and detached from the base and the supports, its mean deviation from the desired shape is about (“0.49 in (a)” + “0.45 in (b)”)/2 = 0.47 mm. However, when the corrected geometry produced by the solver to prevent further shape/size distortions is printed, the printed corrected (compensated) STL geometry (Fig. 19c,d) is much closer to the original one, and their mutual mean deviation equals (“0.31 in (c)” + “0.2 in (d)”)/2 = 0.255 mm. Comparing these two deviations, one can conclude that the corrected surface mesh allows for a decrease in shape/size distortions of up to 1.8 times without any change of operating conditions. And this is the primary aim of the solver presented here, that is to reach higher precision while using the same 3D printing mode.

## Conclusions

We have developed a custom thermal-structural solver capable of modeling the 3D printing of metal parts in both the elastic and elastoplastic zones of the strain–stress curve. The solver is written purely in C#, which is generally considered a high-performance language producing managed code, allowing for system resource management and making software development more focused on implementation of physics and numerical methods rather than on low-level prototyping. The solver was written from scratch and does not rely on any existing libraries for numerical simulation and linear algebra.

The solver’s physical model is based on a Lagrangian approach, wherein the continuum is modeled as a set of points distributed in the volume and connected to their direct neighbors via thin rods or links along which thermal and mechanical interaction can be distributed. To model the elastoplastic behavior of the material when thermomechanical stress exceeds the material’s yield strength, we propose a differential approach to directly integrate strain along each link in time. The method of solution implemented in this solver has proven its effectiveness and precision.

The solver has been evaluated in four different cases and demonstrates that the sacrifice of performance is not significant. The solver has been found to be approximately 1.6 times faster than the Ansys suite of thermal-structural FEM solvers. However, the complexity of the deformable mesh used by the solver makes the geometry pre-processing stage five times slower than the Ansys mesher.

The results produced by the solver have been compared to real-life 3D-printed parts and the relative coincidence is about 98–99.9%, depending on the size of the printed part. Large deviations of 2% for small parts (like the lattice) are caused by the reaching of the 3D scanner precision limit. Larger predicted deformations of printed parts (like the impeller) will reach about 99.9% match to what was actually printed in the LPBF machine.

The results of simulation of 3D-printing of simple lattice and complex turbine geometries show that the simpler the model is, the less its deviation from the original desired design to be printed. Models of size 10–15 [mm] do not require significant shape compensation.

Larger printed parts, in contrast, suffer from distortions. When printing an impeller of 40 [mm] in diameter, its relative size deviation from the reference desired shape is about 1.2%. Larger models will even increase this discrepancy. Therefore, the solver has a functionality to compensate distortions of printed parts.

The primary feature of the solver lies in its ability to predict the deformation of the printed part both before and after cutting off the base. Subsequently, it automatically constructs a corrected STL geometry, which, upon printing, effectively minimizes and compensates for shape and size deviations from the original desired mesh. Extensive real-life and virtual tests have confirmed the solver’s capability to reduce shape distortions by approximately 1.8 times.

In summarizing everything together and closely examining the works of other authors in the field of LPBF simulations, it is essential to identify both the similarities of the presented model to existing ones and describe the differences. This method of LPBF process simulation in our work employs some known speed-up techniques, including voxel meshing, combining physical micro-scale deposited layers into a super-layer to reduce mesh size, utilizing an effective heat source based on analytical estimation of total thermal energy applied to the heated volume per unit time, and layer-by-layer activation of mesh elements according to deposition and processing time. Despite these known techniques, our model introduces novel ideas that significantly distinguish it from existing commercial LPBF simulation frameworks. Let us list them below.

- Firstly, we avoid using the FEM approach to convert the physical problem into the mathematical one. Instead, we employ a combination of the Lagrangian deforming mesh of linked material points and a 1D finite difference approach on a regular mesh to efficiently solve the coupled thermal-structural problem without compromising accuracy and speed. Since we do not consider tensor variables in this particular context, all the mechanical interactions are confined to relaxing 1D beams. Consequently, the solver can work with the principal components of strain and stress tensors as scalar variables. This simplifies the computational process and enhances efficiency.
- Secondly, unlike FEM, we avoid constructing a global matrix of algebraic equations, significantly reducing the required RAM. Instead, we adopt a relaxation technique applied sequentially to each elastoplastic link that constitutes the mesh. This approach allows us to achieve shorter simulation times compared to conventional FEM solvers. In our presented model, the simulation time is considerably reduced, demonstrating superior efficiency. Moreover, MIS- based frameworks have also achieved remarkably fast simulation times (in typical LPBF test cases) compared to complete, uncompromised FEM simulations. As a result, we believe our model’s performance is comparable to that of modern approaches, even though a direct comparison is not feasible.
- Thirdly, we present a novel method to compute the nonlinear elastoplastic behavior of materials under thermal loads, which differs from the conventional flow-based theory of hardening. While the model's formulation relies predominantly on intuition and lacks a mathematically rigorous proof of the existence of a unique solution, it consistently yields results of acceptable accuracy when compared to classic FEM solvers.
- Fourthly, all modules of the model, encompassing data input and output, voxelization, meshing, and the solution itself, are entirely implemented in pure C# without reliance on any existing software packages such as Ansys or Abaqus. This characteristic sets the model apart as an independent all-in-one standalone framework, offering self-sufficiency and flexibility in its application.
- Fifthly, the presented model not only predicts the shape distortion of a printed part but also serves as a technique for automatically achieving residual-sensitive pre-morphing of the part’s shape and size before the printing process starts. This morphing process aims to reduce residual stresses and minimize warpage that may occur after the LPBF process and its detachment from the base.

The combination of these five features establishes the developed model as an efficient solution for enhancing the overall quality of 3D-printed parts with varying sizes and shapes using the LPBF method.

### Supplementary Information

Supplementary Information 1.

Supplementary Information 2.

## Data Availability

The additional datasets used and/or analysed during the current study are available from the corresponding author on reasonable request.
