# Supplementary material for: Thermal-structural hybrid Lagrangian solver and numerical simulation-based correction of shape deformation of stainless-steel parts produced by laser powder bed fusion
Source: Sci Rep. 2023 Oct 16;13:17535. doi: 10.1038/s41598-023-43968-0 (PMC10579232; doi:10.1038/s41598-023-43968-0)
Supplement: Supplementary file 2 — Supplementary Information 2. [file 41598_2023_43968_MOESM2_ESM.docx]

## Supplementary Materials

The following supporting information can be downloaded at “Supplementary Materials” file (S1) presents a dataset of supplementary materials for Figures 11 and 12 of the current manuscript that help to visually compare simulated and real-life printed and scanned geometries and their deformations before and after cutting off the base.

**Suplementary file (S1) for manuscript titled –**

**Thermal-Structural hybrid Lagrangian Solver and Numerical Simulation-Based Correction of Shape Deformation of Stainless-Steel Parts Produced by Laser Powder Bed Fusion**

Ilya Tsivilskiy^1^, and Igor Shishkovsky^2, *^

^1^Kazan Federal University, Kazan, 420008, Russian Federation
^2^Skolkovo Institute of Science and Technology, Center for Materials Technologies, Moscow, 121205, Russian Federation
[^*^I.Shishkovsky@skoltech.ru](mailto:*I.Shishkovsky@skoltech.ru); [shiv@fian.smr.ru](mailto:shiv@fian.smr.ru)

This archive file (Lattice.zip) contains a set of supplementary materials for Figures 11 and 12 of the current manuscript that help to visually compare simulated and real-life printed and scanned geometries and their deformations before and after cutting off the base.

The list of files is as follows:

- lattice.stl is the original geometry to print out, its dimensions are provided in millimeters;
- lat_printed.stl is the real-life printed and scanned geometry attached to the base, its dimensions are in meters;
- lat_sol_printed.stl is a virtually printed (simulation with consequent shape deformation) geometry attached to the base, its dimensions are in meters;
- lat_printed_cutoff.stl is the real-life printed and scanned geometry detached off the base (except its central leg), its dimensions are in meters;
- and lat_sol_printed_cutoff.stl is a virtually printed geometry detached off the base (except its central leg), its dimensions are in meters.

To make the comparison more convenient for the reader, we provide complete ParaView State Files (*.pvsm) that can be easily loaded in ParaView (version 5.9 or higher) via the following sequence of actions: “File – Load State – Navigate to pvsm file location – Search Files Under Specified Directory – Only use files in data directory - Ok”. Once opened, the listed STL models can be observed in 3D space. compare_printed.pvsm is the comparison of virtually and real-life printed models attached to the base, and compare_printed_cutoff.pvsm is the comparison of virtually and real-life printed models cut off the base.

Archive content is possible for downloading by direct link - <https://box.skoltech.ru/index.php/s/rbvDAS8etVpUAbr>
